# Supplementary material for: Engineering unsymmetrically coordinated Cu-S1N3 single atom sites with enhanced oxygen reduction activity
Source: Nat Commun. 2020 Jun 16;11:3049. doi: 10.1038/s41467-020-16848-8 (PMC7297793; doi:10.1038/s41467-020-16848-8)
Supplement: Supplementary file 1 — Description of Additional Supplementary Files [file 41467_2020_16848_MOESM1_ESM.pdf]

Description of Additional Supplementary Files

File Name: Supplementary Movie 1
Description: In-situ ETEM characterization of S-ZIF-8

File Name: Supplementary Movie 2
Description: In-situ ETEM characterization of pure ZIF-8.
