# Supplementary material for: Engineering unsymmetrically coordinated Cu-S1N3 single atom sites with enhanced oxygen reduction activity
Source: Nat Commun. 2020 Jun 16;11:3049. doi: 10.1038/s41467-020-16848-8 (PMC7297793; doi:10.1038/s41467-020-16848-8)
Supplement: Supplementary file 3 — Supplementary Information [file 41467_2020_16848_MOESM3_ESM.pdf]

Supplementary Information for

Engineering unsymmetrically coordinated Cu-S_1N_3 single atom sites with enhanced oxygen reduction activity

Shang *et al.*

Supplementary Figures

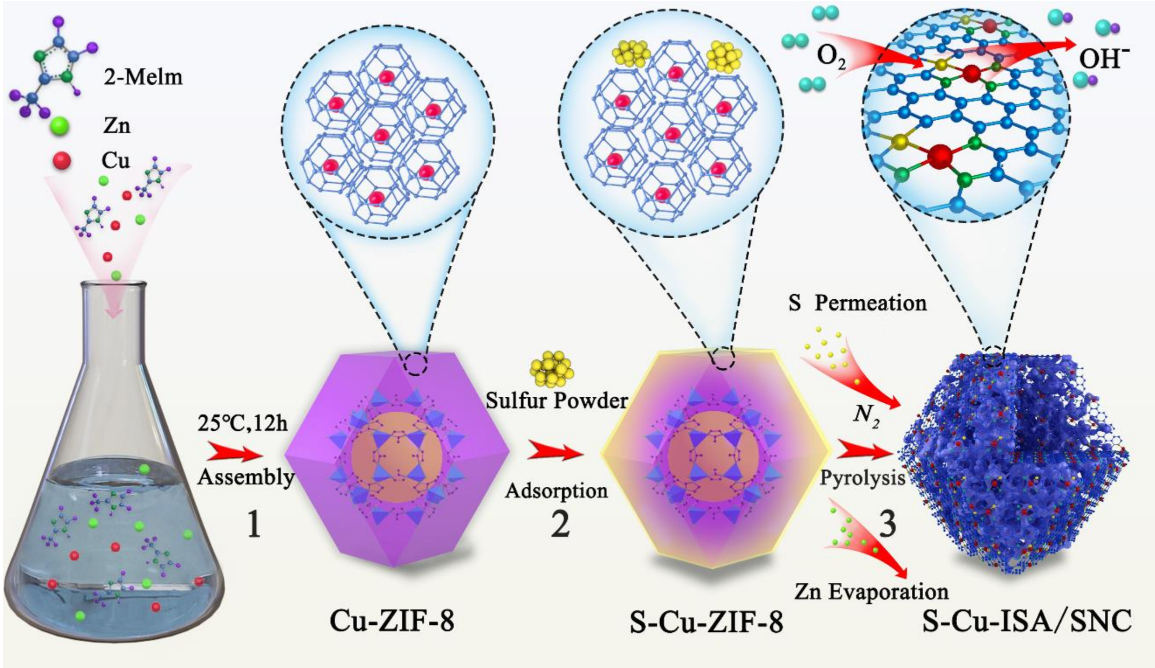

Supplementary Fig. 1. Schematic illustration for the preparation of S-Cu-ISA/SNC. Step one: the self-assembly process for the formation of ZIF-8 together with the encapsulation of Cu cations. Step two: the adsorption of sulfur on the surface of Cu-ZIF-8 powder. Step three: the pyrolyzation of the S-Cu-ZIF-8 to obtained the final S-Cu-ISA/SNC sample.

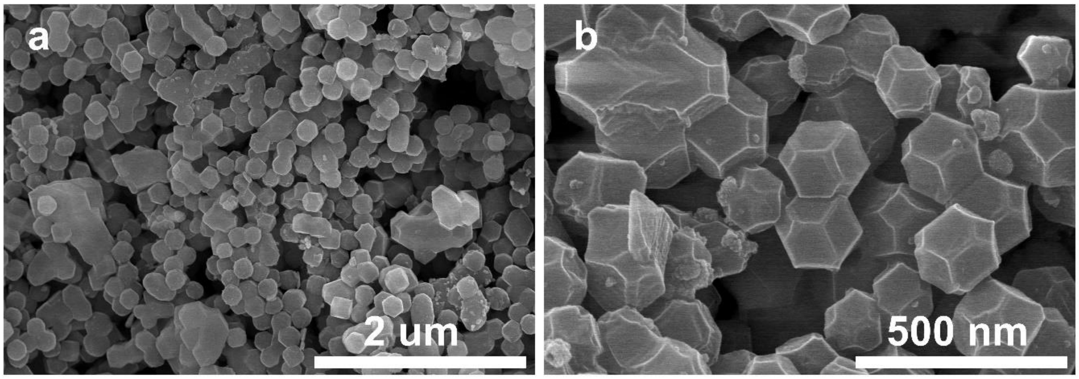

Supplementary Fig. 2. SEM images of the S-Cu-ZIF-8 sample. (a) Low magnitude and (b) high magnitude SEM images. The raw sulfur powder can be dissolved in carbon tetrachloride mixed solution to ensure sulfur species adsorb on MOF particles. In the SEM images, it was observed that the sulfur was absorbed on the surface of Cu-ZIF-8 powder with small size (even below 10 nm). Besides, some large sulfur particles with size of several handred nanometers were also detected.

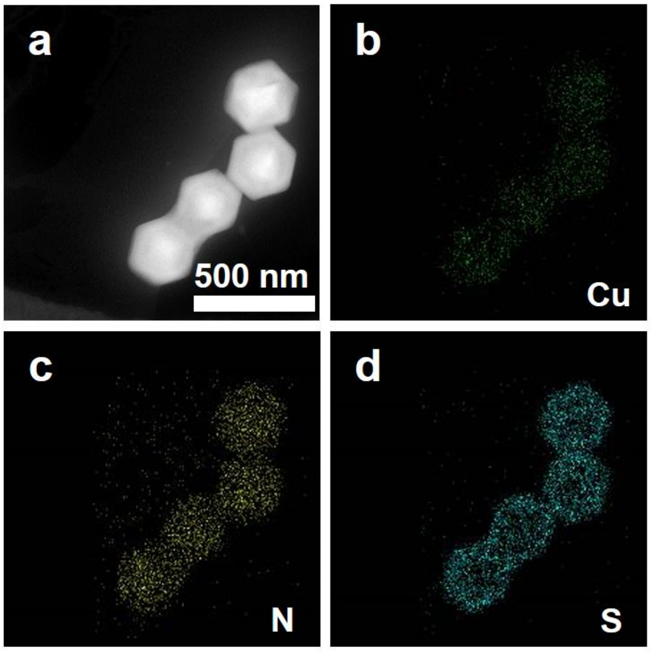

Supplementary Fig. 3. Morphology characterizations of S-Cu-ZIF-8. (a) TEM image and (b-d) Corresponding EDS mapping images of Cu, N and S for S-Cu-ZIF-8, revealing that the sulfur was absorbed on the surface of Cu-ZIF-8 powder.

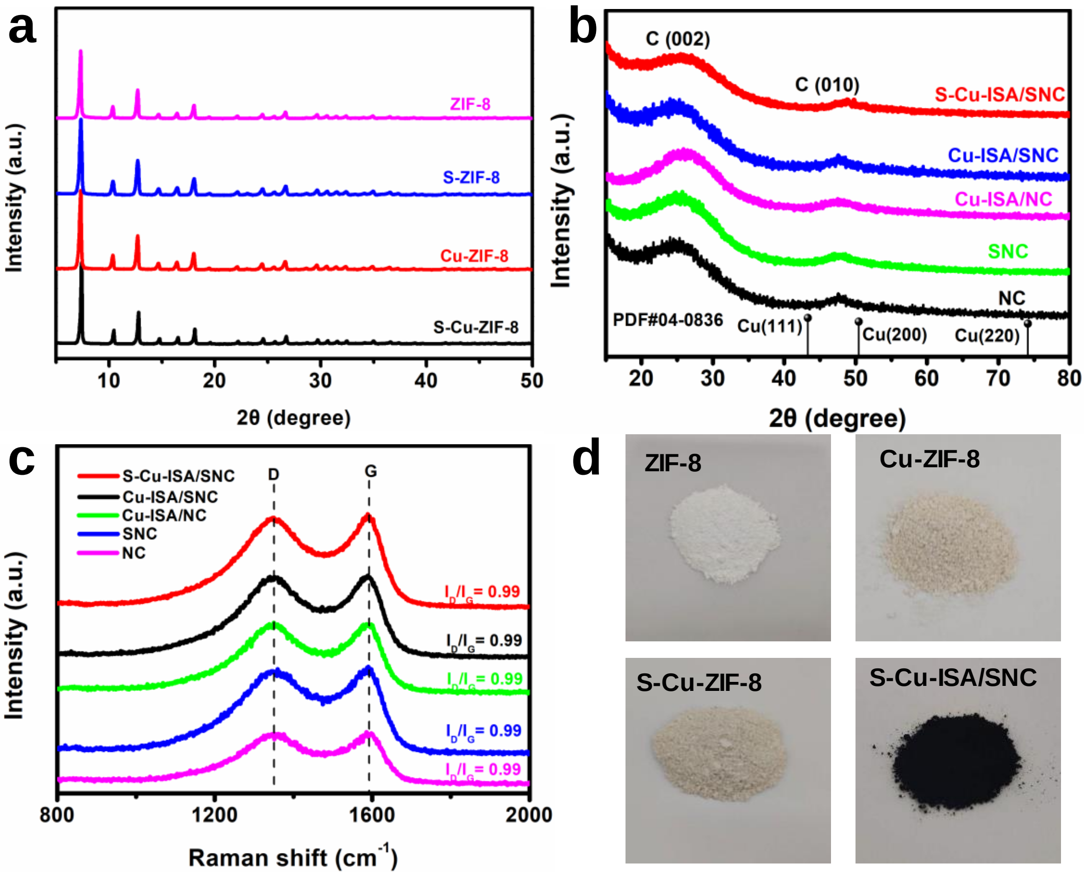

Supplementary Fig. 4. Characterizations of the samples before and after pyrolysis. (a) XRD patterns of pure ZIF-8, S-ZIF-8, Cu-ZIF-8 and S-Cu-ZIF-8. (b) XRD patterns of NC, SNC, Cu-ISA/NC, Cu-ISA/SNC and S-Cu-ISA/SNC. (c) Raman spectra of NC, SNC, Cu-ISA/NC, Cu-ISA/SNC and S-Cu-ISA/SNC. In all samples, only two characteristic peaks of carbon at 1592 cm^{-1} (D band, disordered/defectivecarbon) and 1350 cm^{-1} (G band, graphitic carbon) were detected.[1-3] The intensity ratios of the D-band to G-band (I_D/I_G) were calculated to be 0.99, indicating the high graphitization at 950 ℃. (d) Digital photographs of pure ZIF-8, Cu-ZIF-8, S-Cu-ZIF-8 and S-Cu-ISA/SNC, respectively.

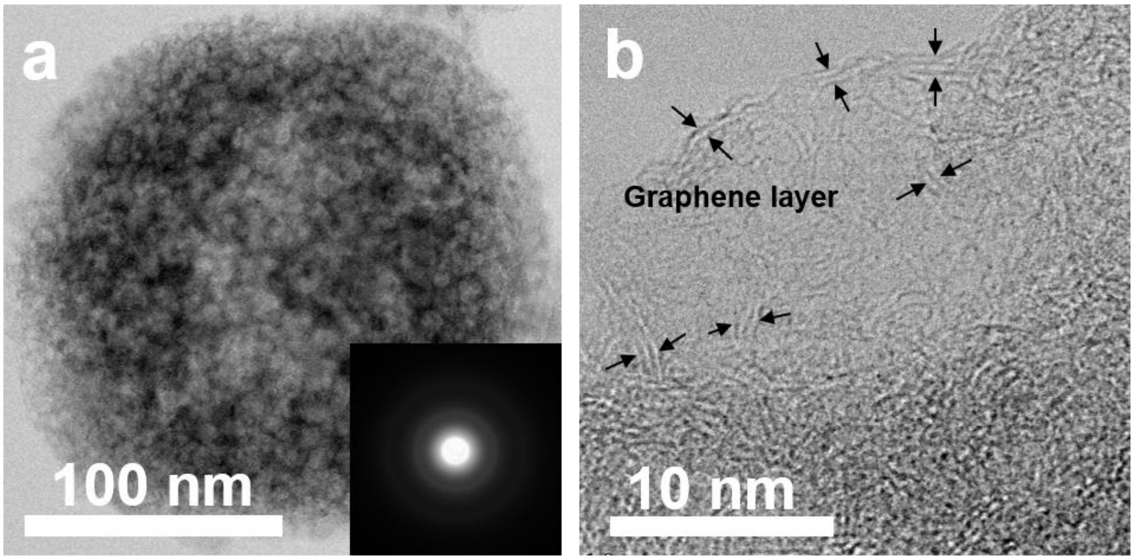

Supplementary Fig. 5. TEM characterizations of S-Cu-ISA/SNC. (a) TEM and (b) HRETM images of S-Cu-ISA/SNC. The inset is the selected area electron diffraction (SAED) pattern of S-Cu-ISA/SNC, which exhibited the poor crystallinity of the carbon frame.

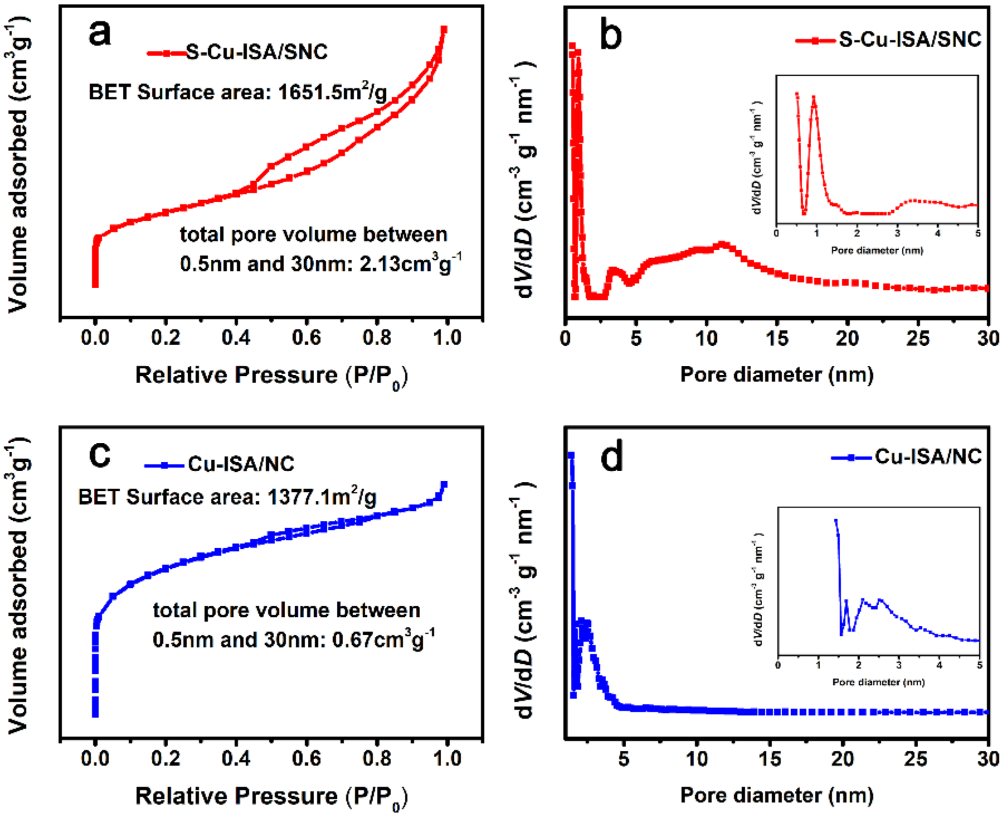

Supplementary Fig. 6. BET surface area and pore size distribution. N_2 adsorption-desorption isotherms (a, c) and pore-size distribution (b, d) of S-Cu-ISA/SNC and Cu-ISA/NC, respectively. N_2 absorption-desorption isotherms (Supplementary Fig. 6a) suggested that the S-Cu-ISA/SNC owned fairly high specific surface area (1651.5 m^2/g). The mesoporous and microporous characteristics of S-Cu-ISA/SNC were analyzed by nitrogen adsorption measurements (Supplementary Fig. 6b). The pore size distribution was analyzed by the quenched solid density functional theory (QSDFT) model to calculate the micropore size and mesopore size separately.[4-5] The pore volume for S-Cu-ISA/SNC was 2.13 cm^3 g^{-1}, much larger than that of Cu-ISA/NC (0.67 cm^3 g^{-1}). The S-Cu-ISA/SNC showed abundant mesopores with a large size distribution from 3 to 30 nm. Furthermore, micropore structures were also identified for S-Cu-ISA/SNC with pore size about 1 nm, which should be attributed to the carbonization of ZIF-8 and evaporation of metallic zinc. The Cu-ISA/NC showed mesopores with size distribution from 2 to 5 nm. These data revealed that the addition of sulfur significantly increased the surface area and also changes the pore structure of carbon substrates.[6-8]

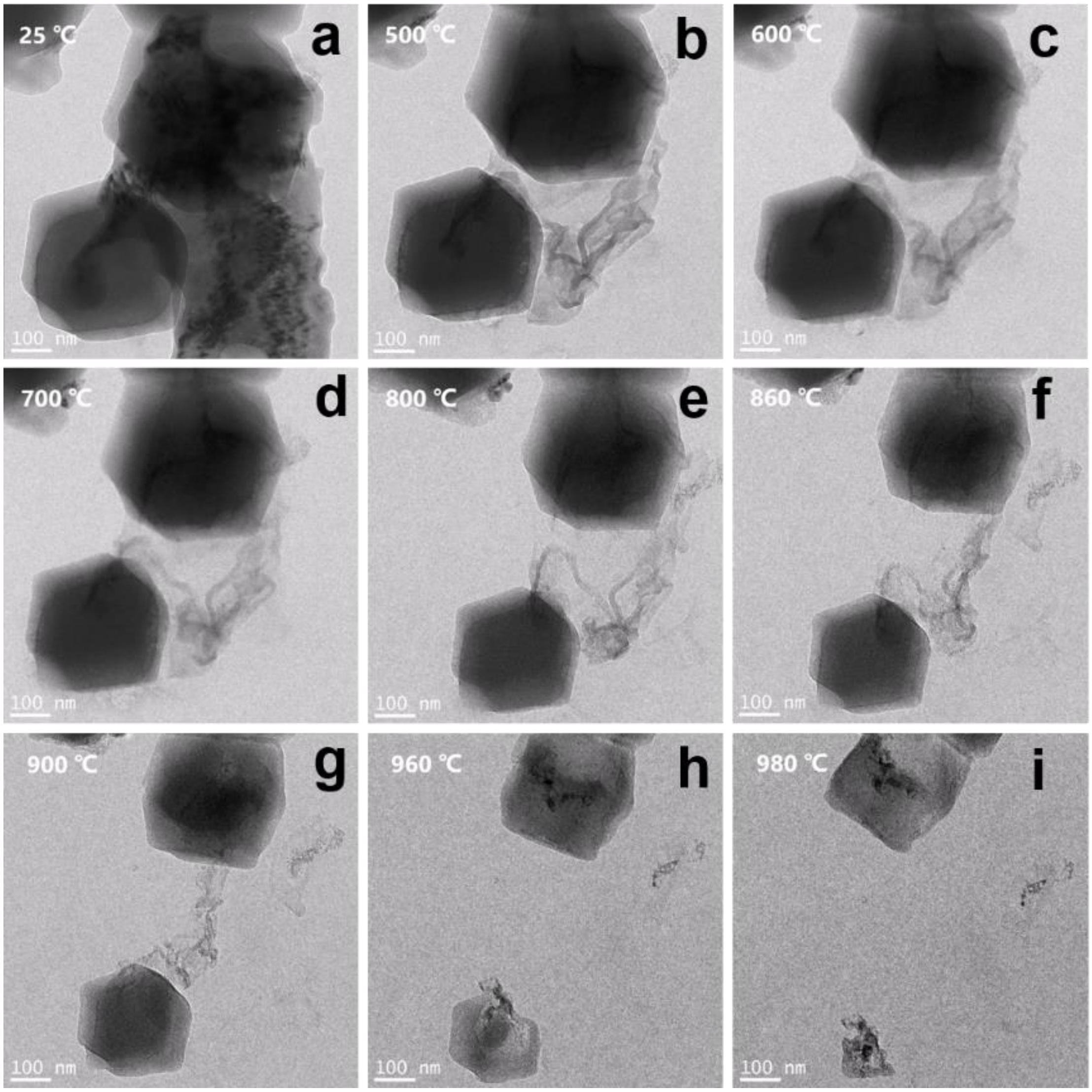

Supplementary Fig. 7. *In-situ* ETEM characterization of S-ZIF-8. The characterization was performed at various temperatures under Ar atmosphere from Supplementary Movie 1. The sample was obtained by combining ZIF-8 powder with excess sulfur species. We found that the ZIF-8 particles in the field of view became shrunken following the increasing of temperature. Especially, the smaller particle even disappeared when the temperature was up to 980℃. The *in-situ* test elucided the remarkable etching effect of the endosmic sulfur for the pyrolized ZIF-8 frameworks.

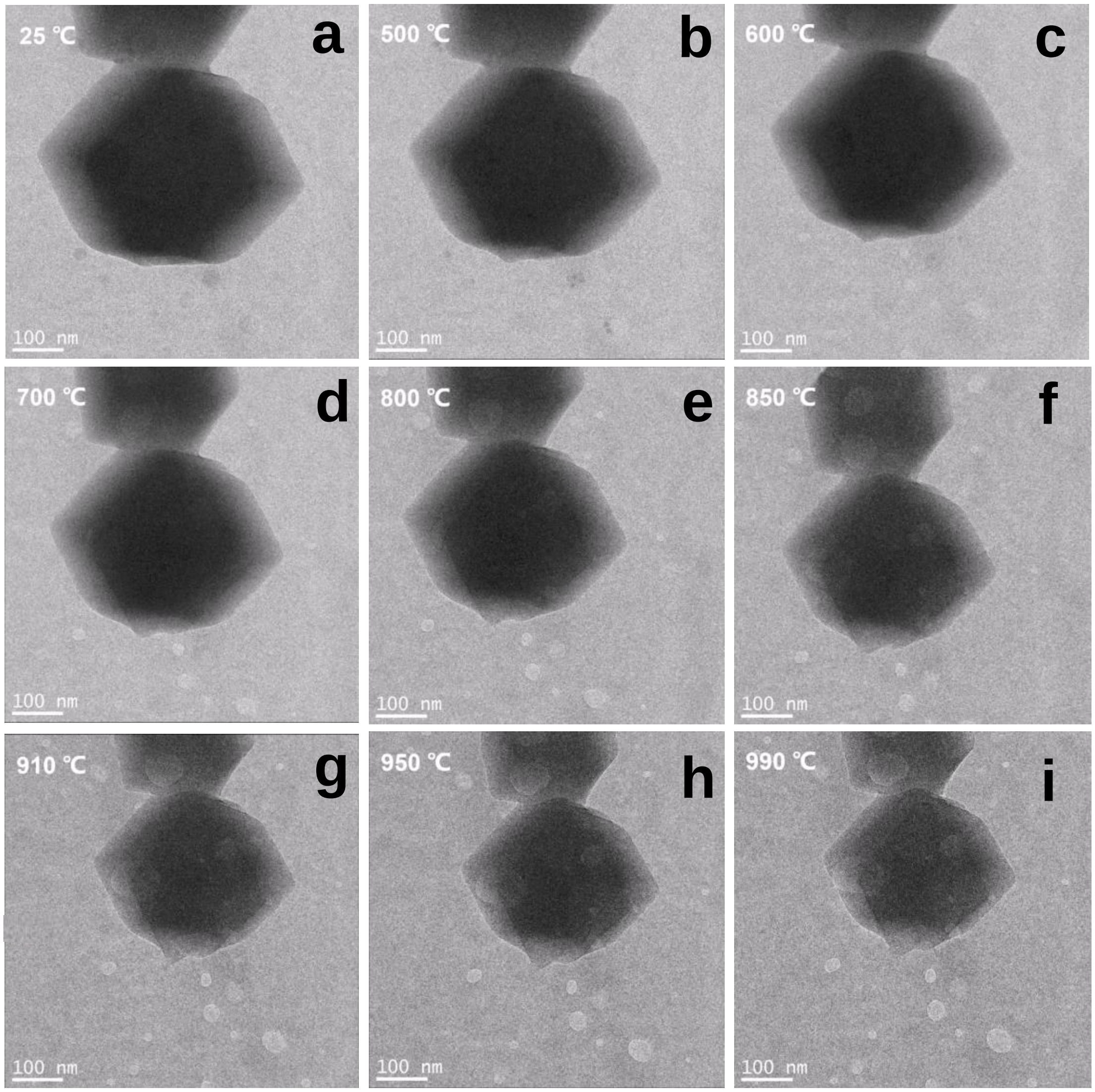

Supplementary Fig. 8. *In-situ* ETEM characterization of pure ZIF-8. The characterization was performed at various temperatures under Ar atmosphere from Supplementary Movie 2. The ZIF-8 nanoparticles was encapsuled in the chip without sulfur. During the increasing of tempereture, the particles became smaller. While they maintain their size and shape after the temperature 910 ℃. The size reduction of the ZIF-8 frames was mainly due to the evaporation of Zn species and when most of Zn in the particles was lost, the frameworks kept stable, which was consistent with the previous report.[9]

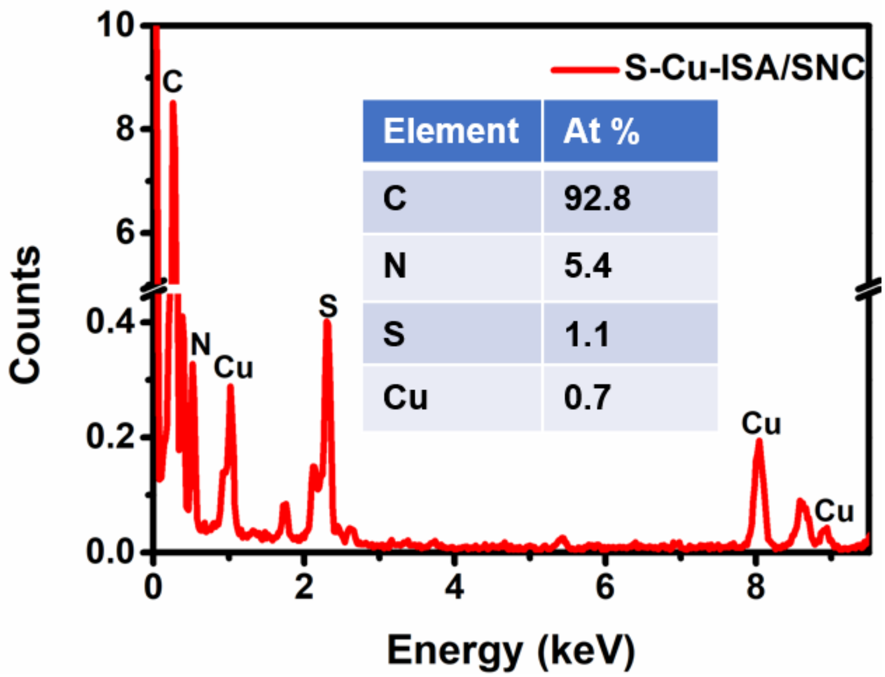

Supplementary Fig. 9. EDS spectrum of S-Cu-ISA/SNC. The element content in S-Cu-ISA/SNC was also labeled in the quantitative EDS spectrum. The actual loading of Cu in S-Cu-ISA/SNC was about 0.7 at %, corresponding to the results of ICP-OES.

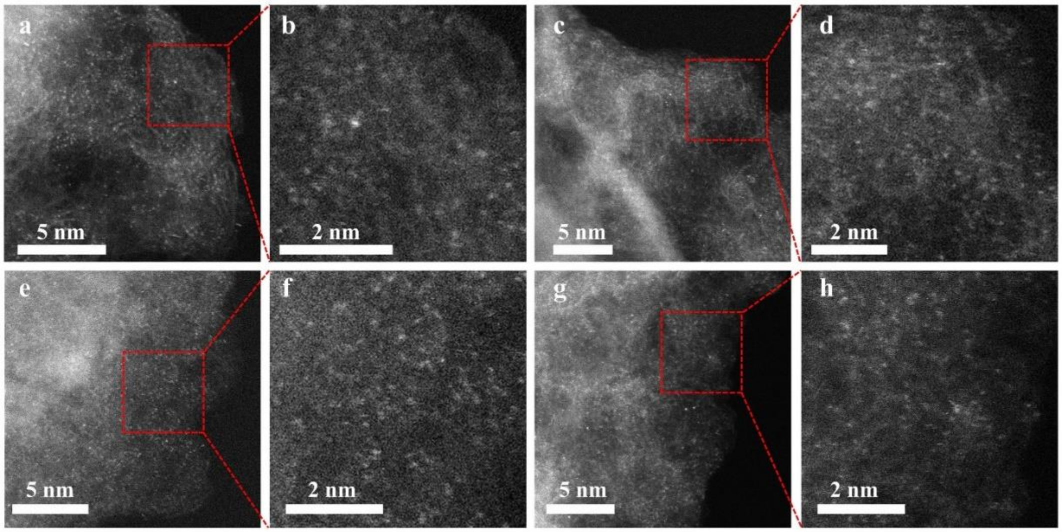

Supplementary Fig. 10. Representative HAADF-STEM images (a, c, e, g) and the enlarged images (b, d, f, h) of Cu SAs at four different areas for the sample S-Cu-ISA/SNC.

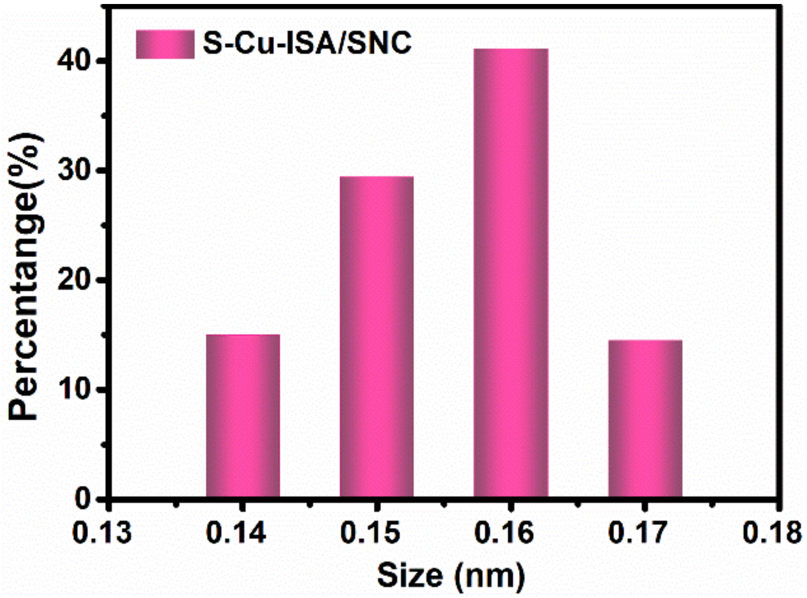

Supplementary Fig. 11. The size distribution of Cu signal detected by EDS-HRTEM. All of Cu species in S-Cu-ISA/SNC is less than 0.20 nm, indicating that Cu exists exclusively in atomic dispersion.

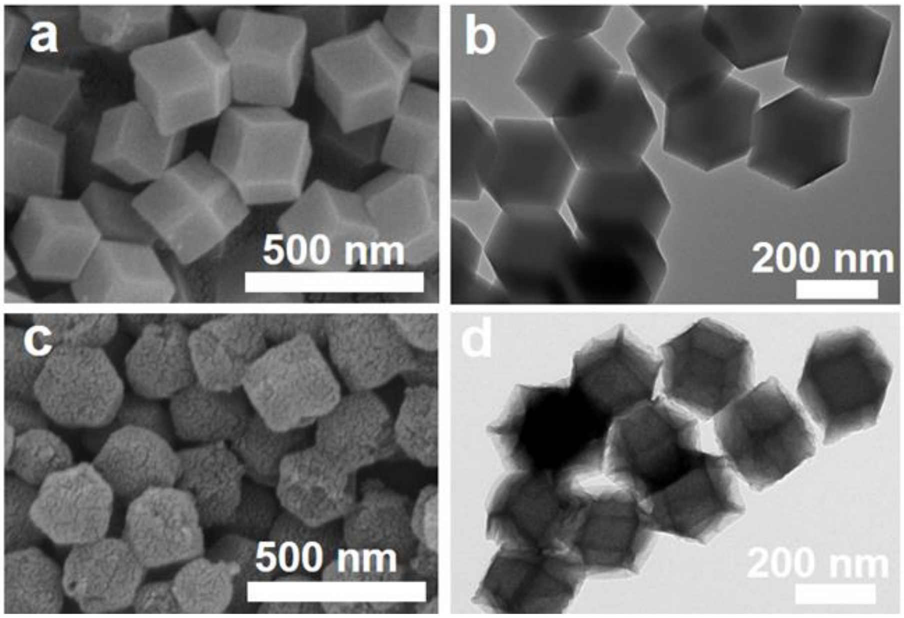

Supplementary Fig. 12. Morphology characterizations of ZIF-8 and pyrolyzed-ZIF-8. (a-b) SEM and TEM images of ZIF-8, (c-d) SEM and TEM images of pyrolyzed-ZIF-8 (NC). The pyrolyzed-ZIF-8 remains the polyhedral shape.

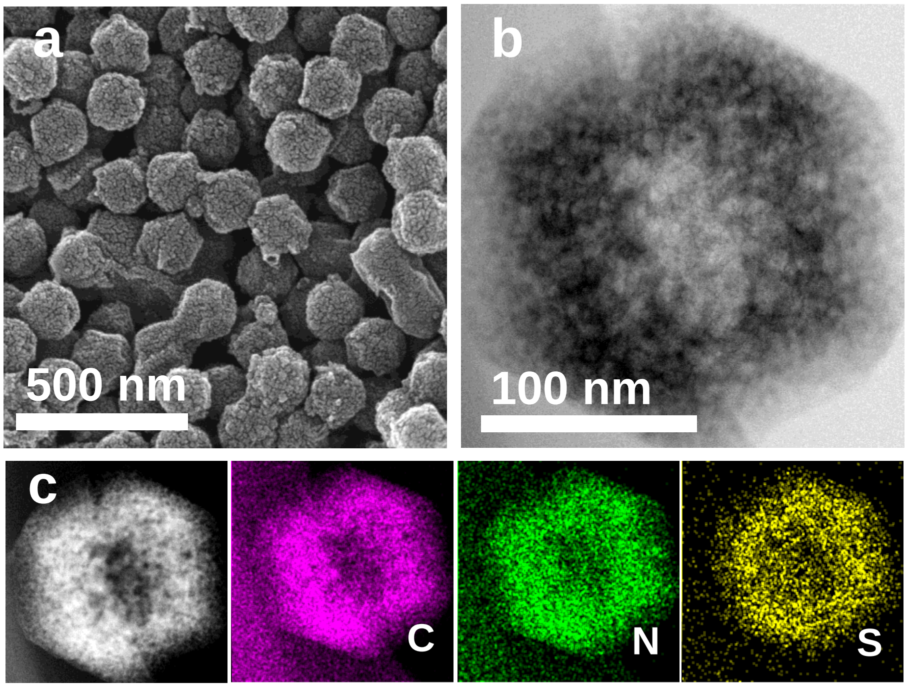

Supplementary Fig. 13. Morphology characterizations of SNC. (a, b) SEM and TEM images, (c) HAADF STEM images and corresponding EDS images of C (pink), N (green) and S (yellow) for SNC.

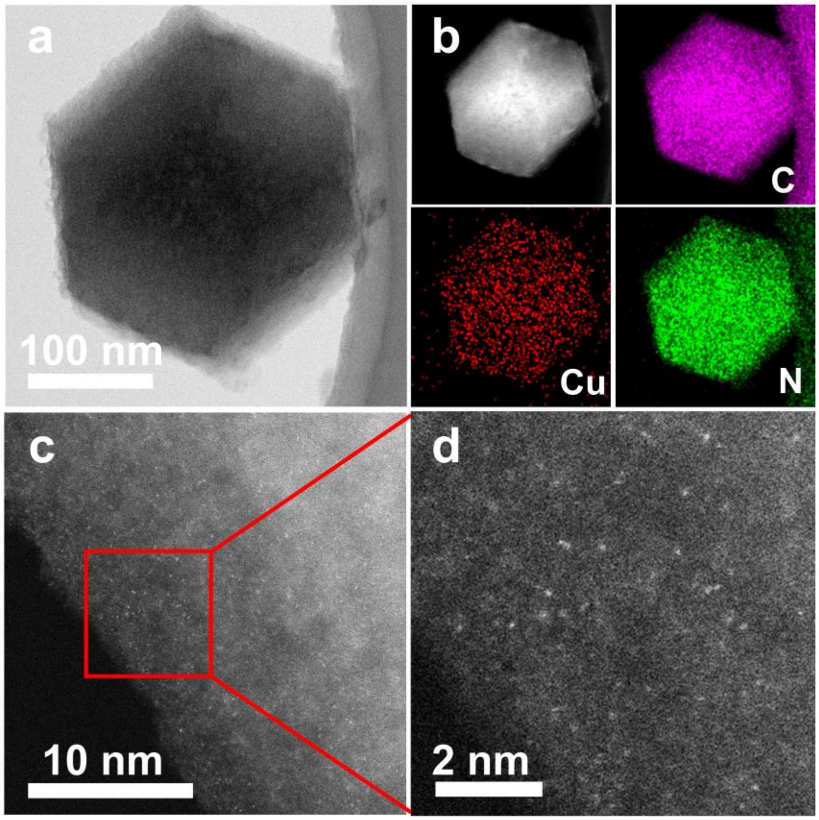

Supplementary Fig. 14. Morphology characterizations of Cu-ISA/NC. (a) TEM images of Cu-ISA/NC (b) HAADF-STEM images and Corresponding EDS mapping images of Cu, C and N for Cu-ISA/NC, revealing the homogeneous distribution of Cu and N on the carbon support. (c) HAADF-STEM image and (d) the enlarged HAADF-STEM images.

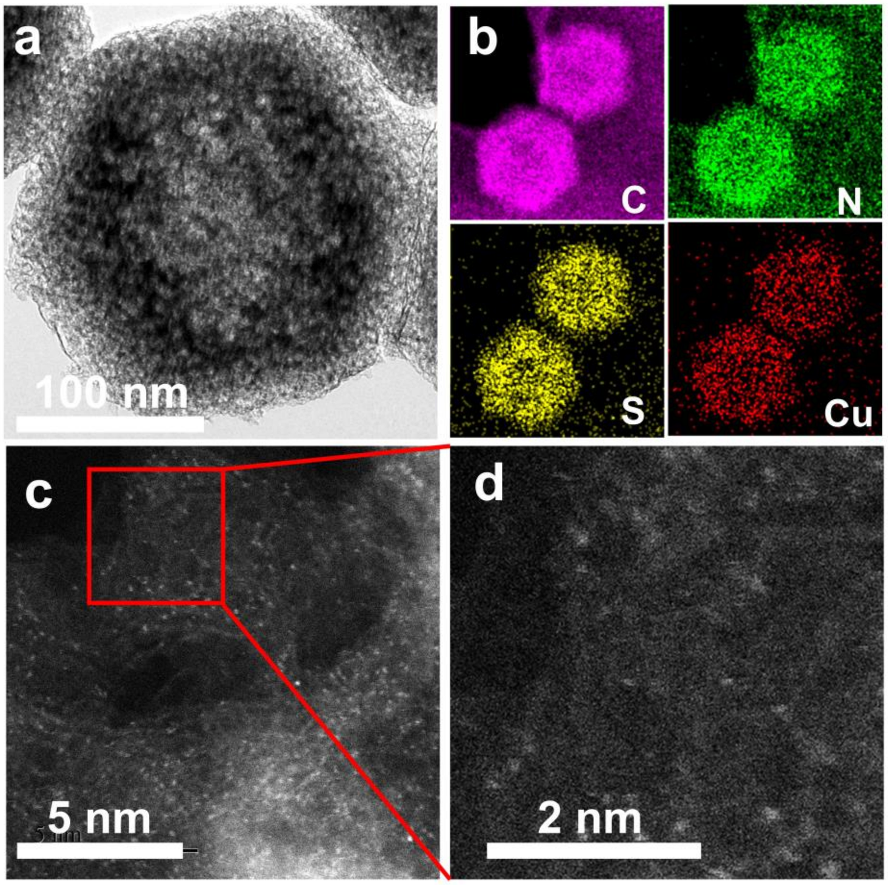

Supplementary Fig. 15. Morphology characterizations of Cu-ISA/SNC. (a) TEM images of Cu-ISA/SNC. (b) EDS images, C (pink), N (green), S (yellow) and Cu (red). (c) HAADF-STEM image and (d) the enlarged HAADF-STEM image.

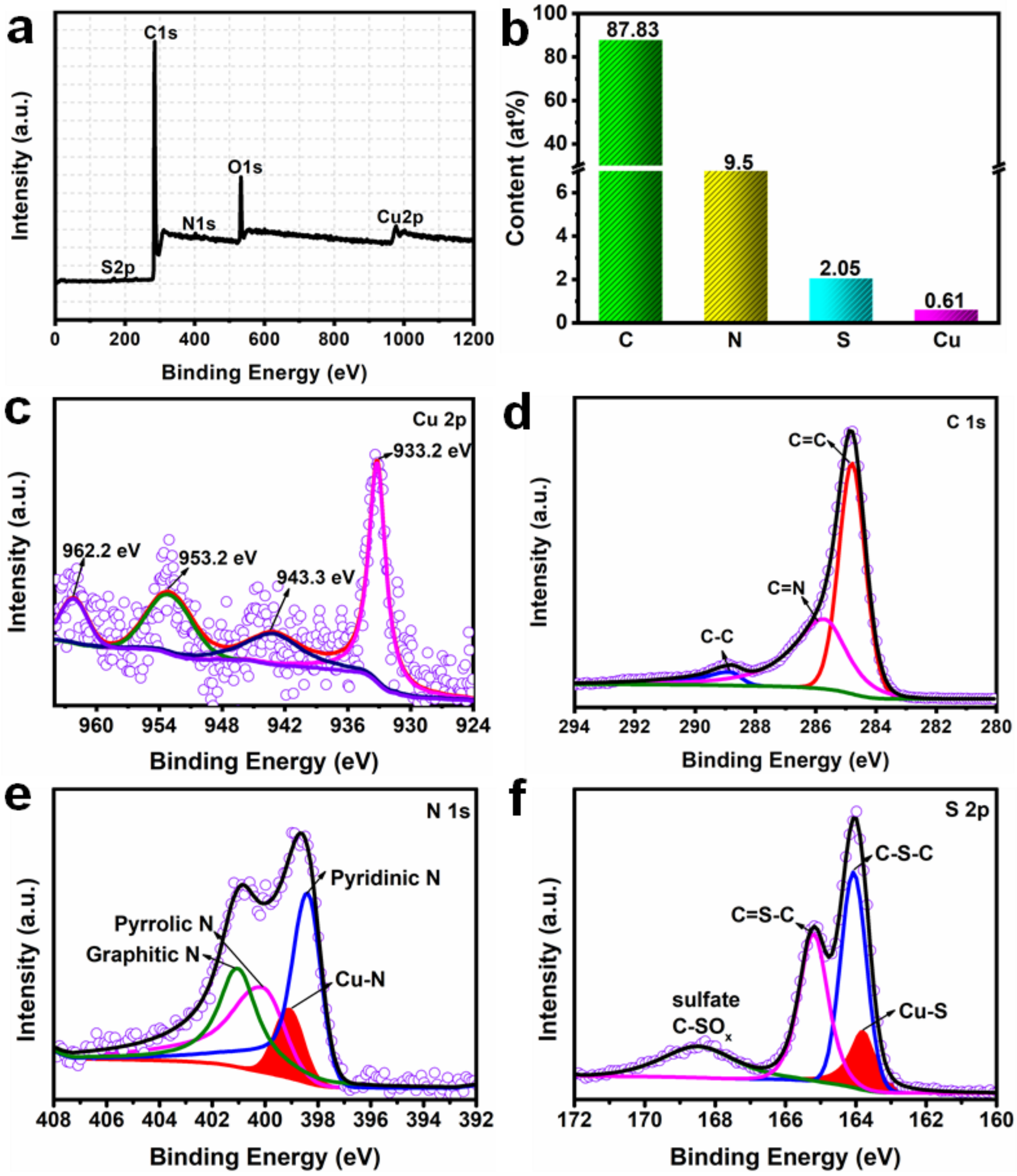

Supplementary Fig. 16. XPS spectra for S-Cu-ISA/SNC. (a) XPS spectra for the survey scan of S-Cu-ISA/SNC. (b) The atomic content percentages of C, N, S and Cu in S-Cu-ISA/SNC measured by XPS analysis. The XPS spectra for the (c) Cu 2p, (d) C 1s, (e) N 1s and (f) S 2p region of S-Cu-ISA/SNC. The XPS survey spectra confirmed the presence of Cu, N, S, and C species, indicating the successful doping of Cu, N and S elements in the carbon support (Supplementary Fig. 16a). The corresponding contents of Cu, N, S, and C species are summarized in Supplementary Fig. 16b. The Cu 2p spectrum (Supplementary Fig. 16c) demonstrated two types of peaks. The characteristic peak of Cu $2p_{3/2}$ located at 933.2 eV situating between Cu^0 (932.4 eV) and Cu^{2+} (934,6 eV),

indicating the ionic $Cu^{\delta+}$ ($0<\delta<2$) nature of Cu in S-Cu-ISA/SNC.[10] The peak at 943.3 eV and 962.2 eV could be ascribed to the satellite peaks.[11] The C 1s XPS spectra (Supplementary Fig. 16d) manifested three peaks at binding energies of 284.8, 285.8 and 288.9 eV, which were assigned to graphitic sp^2 carbon (C=C), carbon coordinated with doped N (C-N), and sp^2 carbon (C-C) bonds, respectively.[12] The N 1s spectra (Supplementary Fig. 16e) was classified to four types of N species, namely, 398.4 (pyridinic N), 399.1 (Cu-N), 400.2 (pyrrolic N) and 401.1 (graphitic N).[13, 14] The S 2p XPS spectra (Supplementary Fig. 16f) show three types of S species. The peak at 168.3 eV assigned to the sulfate species (C-SO_x), while the two peaks at 164.9 eV and 163.6 eV were associated to C=S-C and C-S-C bond, respectively.[15] Most importantly, the characteristic peaks at 163.9 eV, corresponding to the Cu-S bond, were observed, which could stem from the partial replacement of N atoms with S to form the Cu-S bond. All these results indicated that the atomically dispersed Cu possessed typical Cu-N and Cu-S dual coordinating environment.

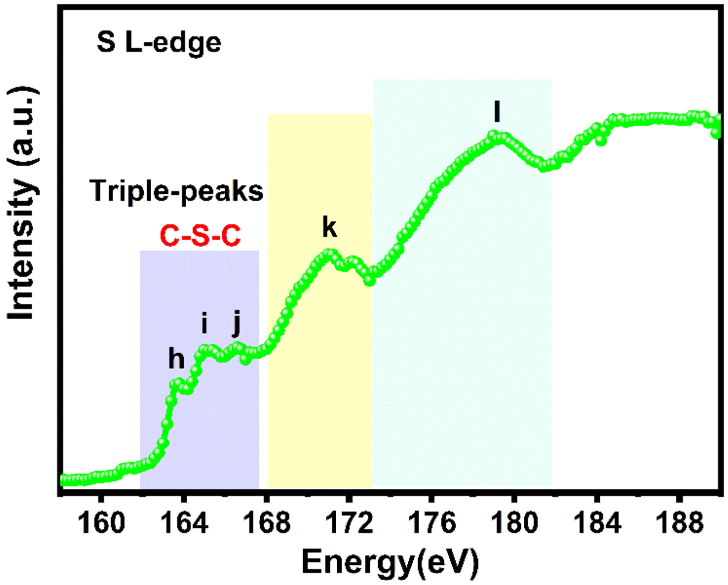

Supplementary Fig. 17. S L-edge XANES spectra of the S-Cu-ISA/SNC. The S L-edge features result from the electronic transitions of 2p electrons to partially filled or empty molecular orbitals.

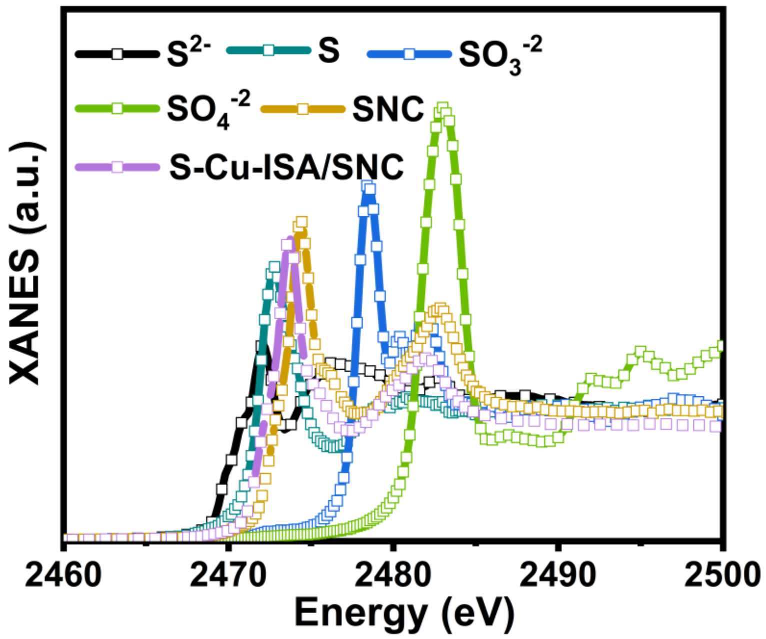

Supplementary Fig. 18. S K-edge XANES spectra of S-Cu-ISA/SNC and reference materials.

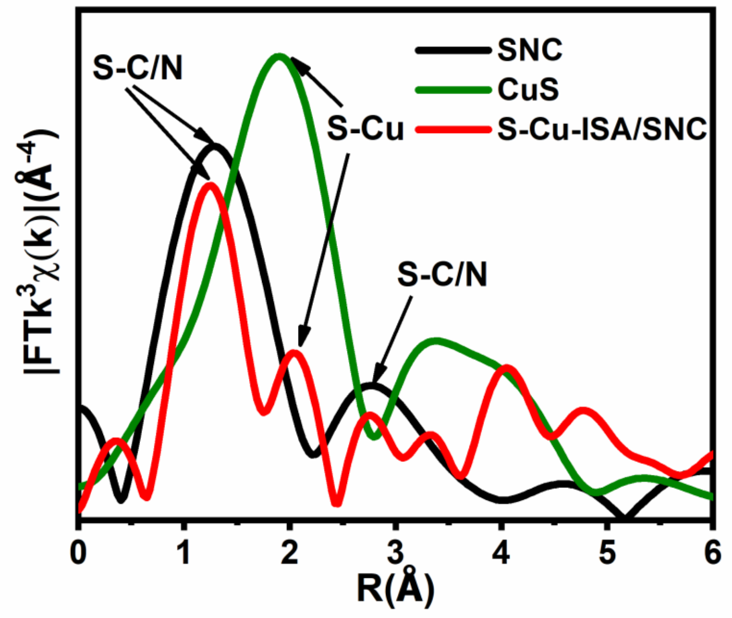

Supplementary Fig. 19. FT magnitudes of the experimental S K-edge EXAFS signals of S-Cu-ISA/SNC, CuS and SNC. It can be observed that, in comparison with CuS and SNC, S-Cu-ISA/SNC shows an additional peak at 2.1 Å, which can be ascribed to the presence of S-Cu nearest-neighboring coordination.

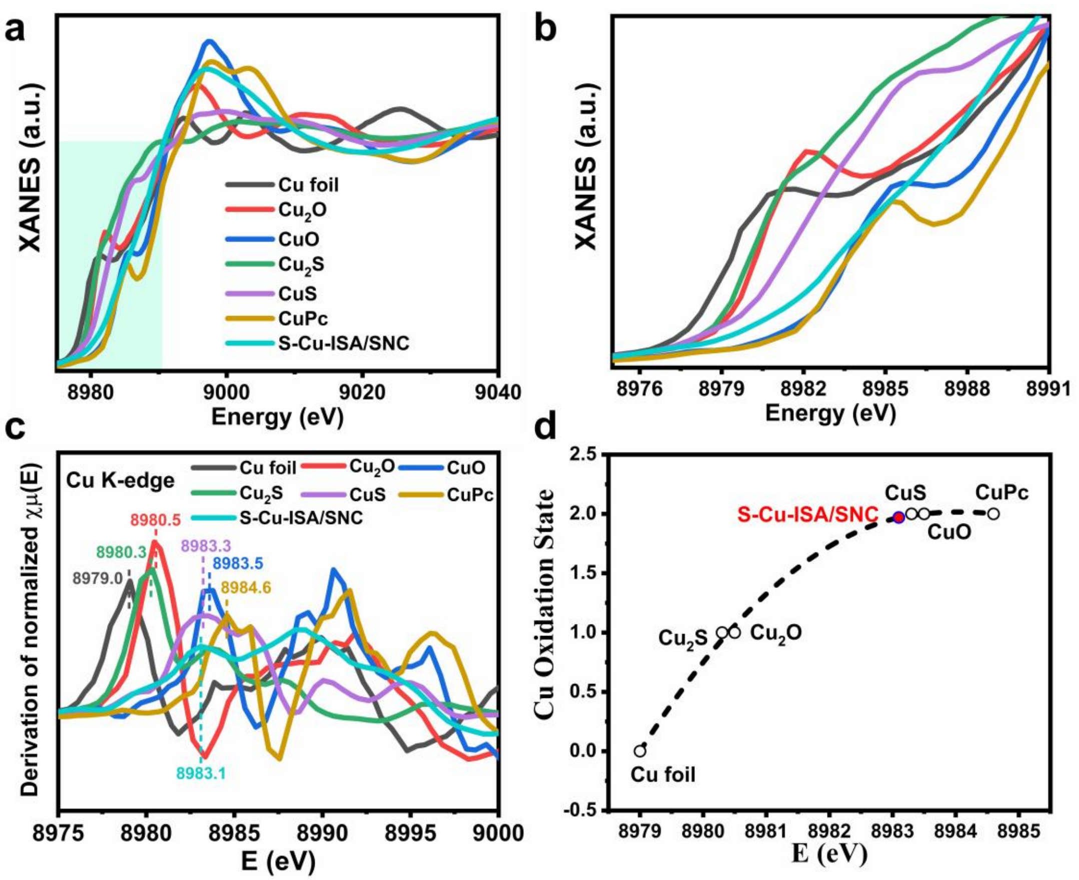

Supplementary Fig. 20. Characterization of Cu oxidation state from XANES. (a-b) The Cu K-edge XANES spectra of S-Cu-ISA/SNC and the references (Cu foil, Cu_2O, CuO, Cu_2S, CuS and CuPc). (c) First-derivative XANES curves of S-Cu-ISA/SNC and the references. (d) Correlation between the Cu oxidation state and the energy position of the XANES spectrum, determined as the first maximum of the first derivative spectrum of S-Cu-ISA/SNC and different copper reference compounds.

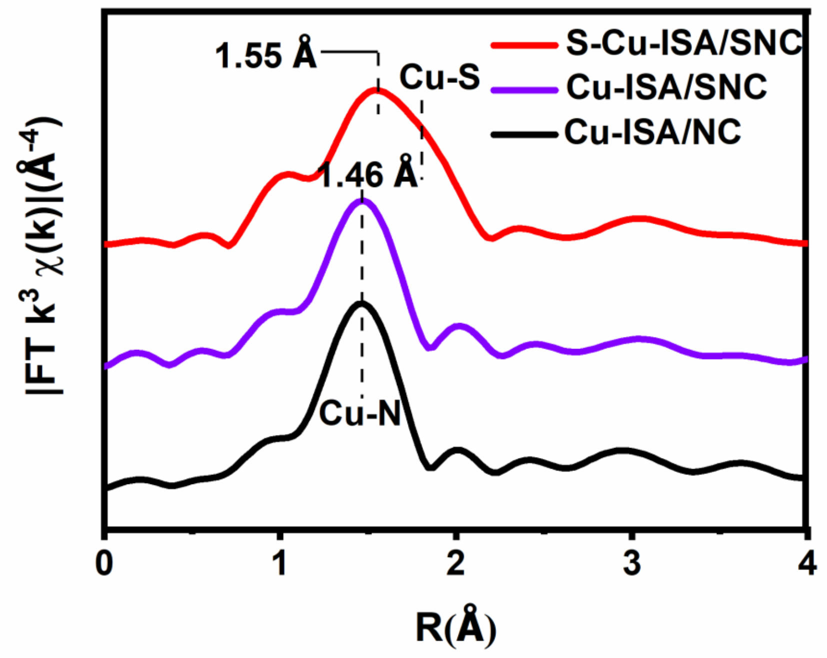

Supplementary Fig. 21. FT-EXAFS fitting curves of S-Cu-ISA/SNC, Cu-ISA/SNC, and Cu-ISA/NC. The Cu-ISA/SNC and Cu-ISA/NC exhibit one obvious FT peak located at 1.46 Å, attributed to the scattering of Cu-N coordination. The Cu-S scattering signal in S-Cu-ISA/SNC induce the shift of Cu-N to 1.55 Å.

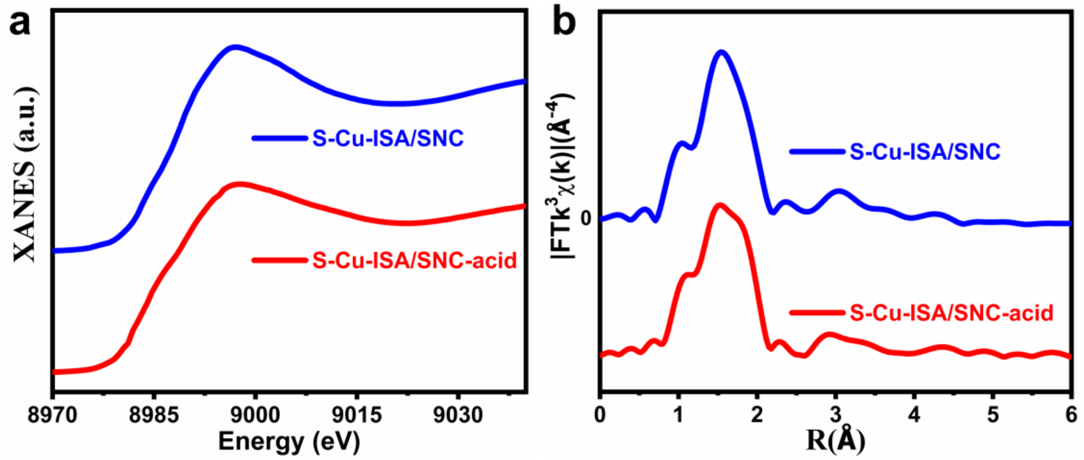

Supplementary Fig. 22. XAS characterizations of S-Cu-ISA/SNC before and afer acid treatment. (a) The XANES spectra and (b) k^3-weighted FT-EXAFS Cu K-edge spectra of S-Cu-ISA/SNC before and after dilute nitric acid treatment. In order to further exclude the formation of copper sulphide species in S-Cu-ISA/SNC, the sample was immersed in dilute nitric acid (HNO_3) solution (1 mol/L) at 60 ℃ for 24 h. Due to the fairly high specific surface area and the hierarchically porous characteristics of carbon based frameworks, the dilute nitric acid solution can thoroughly permeate in the whole structure of the S-Cu-ISA/SNC polyhedron, so that the copper sulphide species can be removed if they exist in the sample. After drying, the acid-treated sample was characterized by XAFS again, which we displayed in Supplementary Fig. 22. It is found that both the XANES (Supplementary Fig. 22a) and EXAFS (Supplementary Fig. 22b) curves are with no obvious change, indicating that the Cu species in S-Cu-ISA/SNC keep the same local atomic structure before and after acid treatment. These results further demonstrate the uniformly isolated Cu species in S-Cu-ISA/SNC and exclude the possible formation of copper sulphide nanoparticles or clusters.

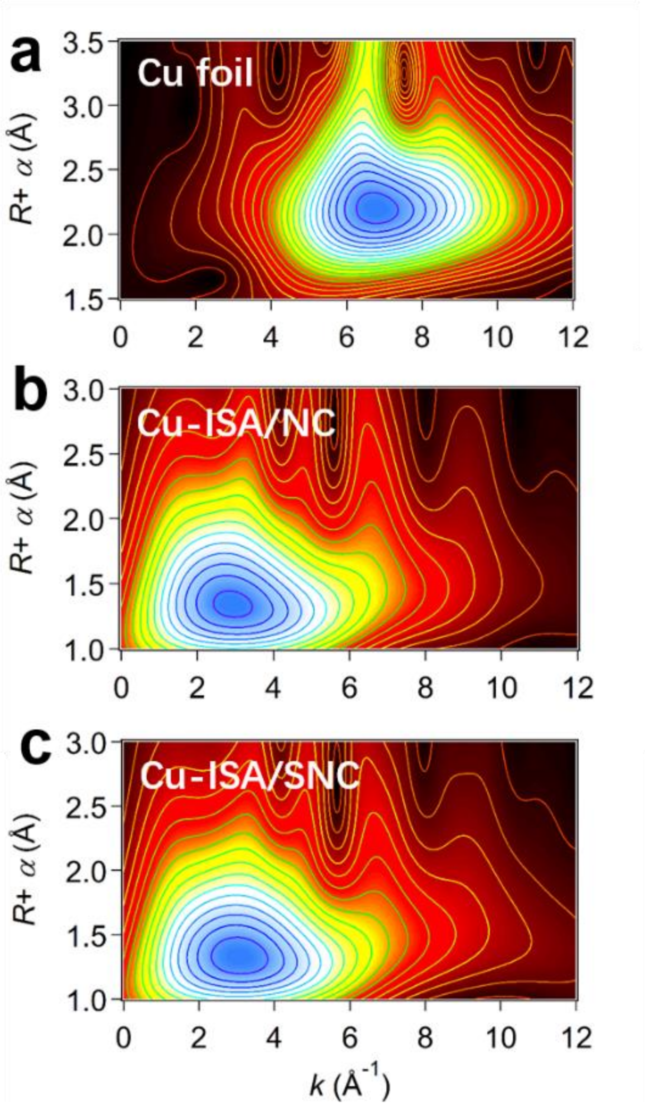

Supplementary Fig. 23. Wavelet transforms for the k^3-weighted Cu K-edge EXAFS signals. (a) Cu foil, (b) Cu-ISA/NC, (c) Cu-ISA/SNC. An obvious disadvantage of FT is that it only has a resolution in Fourier space and not in the space of original data. In EXAFS analysis, the FT magnitude of experimental data provides resolution in the radial distance of neighbouring scatterers. However, the information is lost at the wave number *k* at which the scatterer contributes. Since the position in *k*-space is related to the atomic species of the backscattering atom, important information is lost in the magnitude of the transformed signal. So it is necessary to develop new methods for the analysis of EXAFS, such as WT, which has powerful resolution in both *k* and *R* spaces. WT is thought to be a wonderful supplement for FT.[16,17]

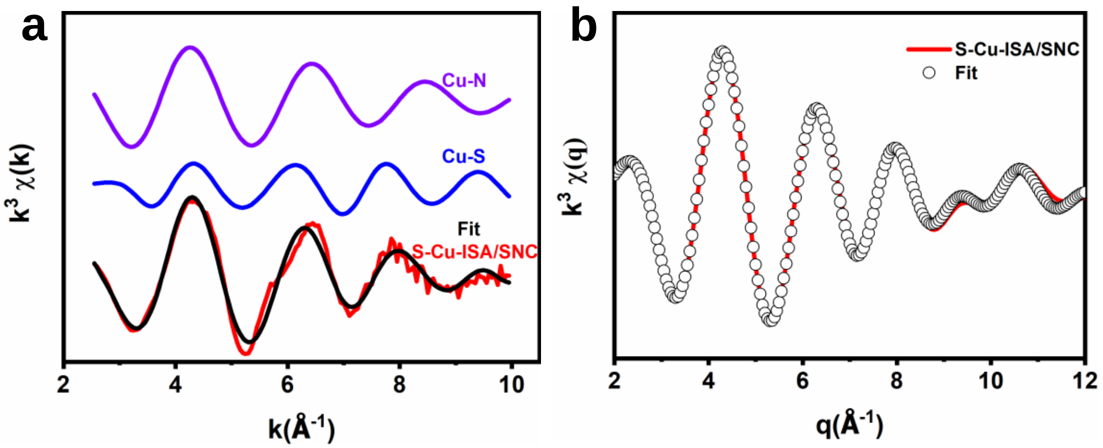

Supplementary Fig. 24. EXAFS fitting of S-Cu-ISA/SNC. (a) the EXAFS fitting curve of S-Cu-ISA/SNC at Cu K-edge. We carried out EXAFS curve-fitting for the first coordination shell of Cu by considering two backscattering paths, including Cu-N and Cu-S. (b) q space fitting curve at Cu K-edge of S-Cu-ISA/SNC. The best-fit structural parameters are listed in Supplementary Table 1.

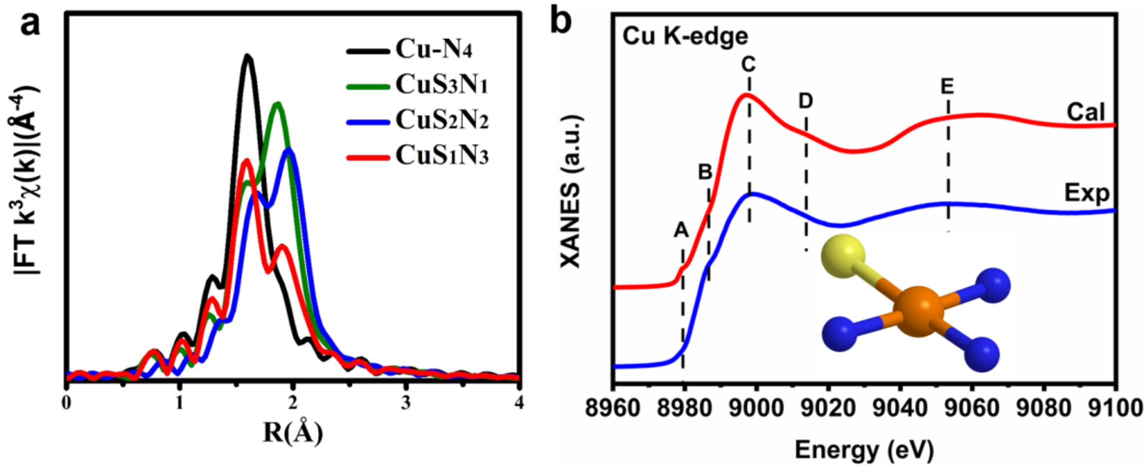

Supplementary Fig. 25. The simulated XAFS spectra. (a) the simulated EXAFS spectra with Cu_1-S_1N_3, Cu_1-S_2N_2, Cu_1-S_3N_1 and Cu_1-N_4 models. (b) the simulated XANES spectrum based on the instead atomic interface structure of Cu-S_1N_3, compared with the experimental spectrum of S-Cu-ISA/SNC. The Cu K-edge theoretical XANES simulations were carried out with the FDMNES code in the framework of real-space full multiple-scattering (FMS) scheme using Muffin-tin approximation for the potential. The energy dependent exchange-correlation potential was calculated in the real Hedin-Lundqvist scheme, and then the spectra convoluted using a Lorentzian function with an energy-dependent width to account for the broadening due both to the core-hole width and to the final state width.[18,19]

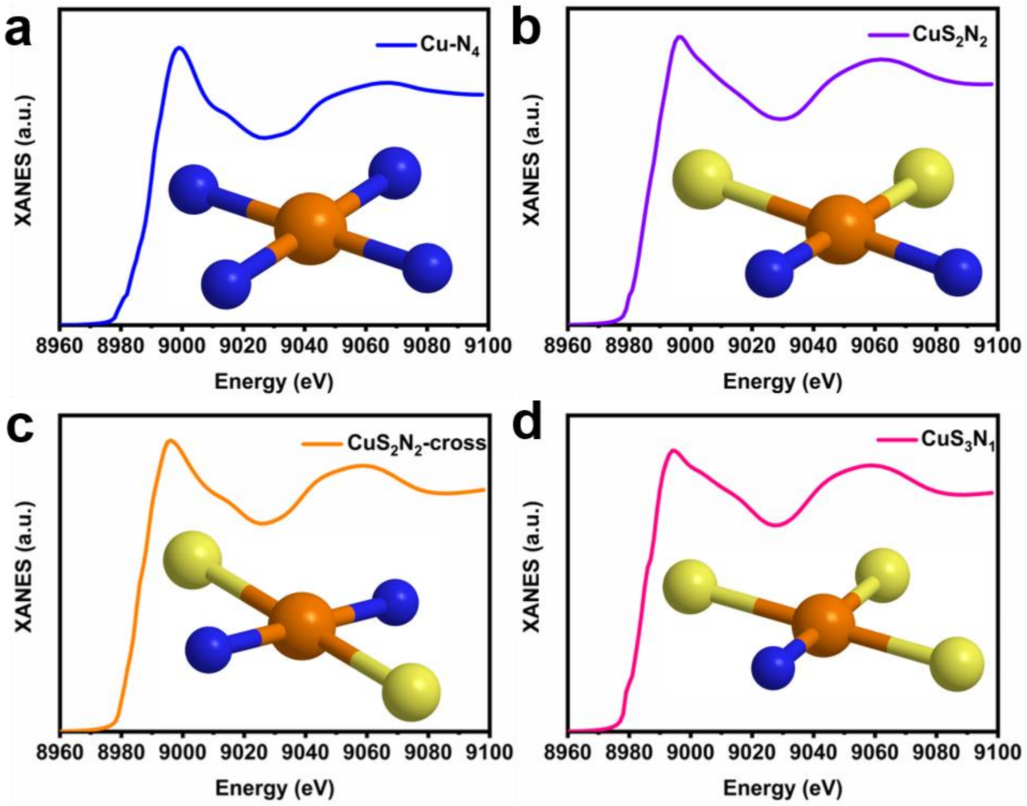

Supplementary Fig. 26. The simulated XANES spectrum based on different atomic interface structures. (a) Cu-N_4, (b) Cu-S_2N_2, (c) Cu-S_2N_2-cross (d) Cu-S_3N_1.

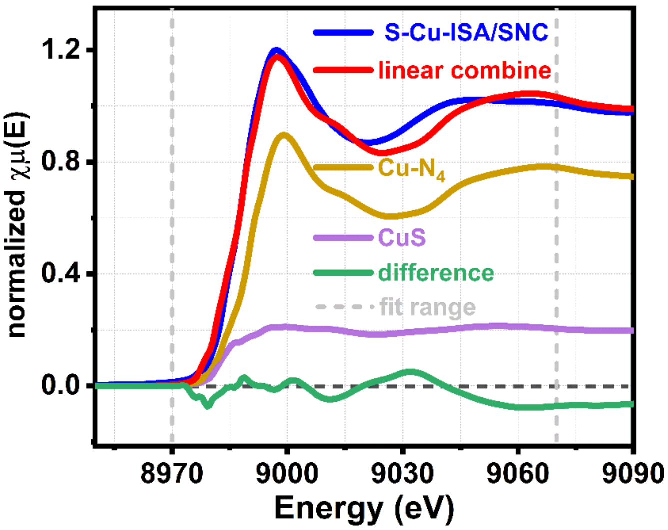

Supplementary Fig. 27. The linear combination fitting of the experimental spectrum of S-Cu-ISA/SNC with the calculated spectrum for CuN_4 and experimental spectrum for CuS. As we know, ATHENA has a capability of fitting a linear combination of standard spectra to an unknown spectra. These fits can be done using normalized μ(E) spectra. The percentage of the three standard components was auto adapted by Athena software, as well as the fitting in Supplementary Fig. 28 and 29. Herein, we claim that it is hard for us to obtain a standard experimental spectrum which is perfectly matched with the CuN_4 model, so a calculated spectrum of CuN_4 (although it may introduce some artefacts) was applied to give indirect evidence of the presence of Cu single atoms coordinated with both N and S.

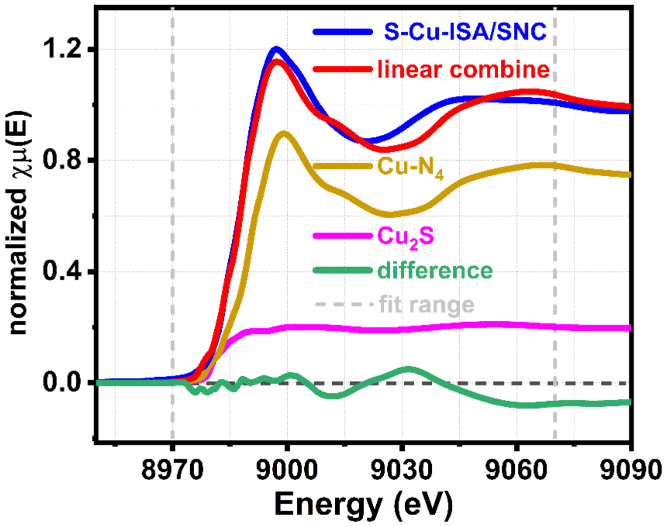

Supplementary Fig. 28. The linear combination fitting of the experimental spectrum of S-Cu-ISA/SNC with the calculated spectrum for CuN_4 and experimental spectraum for Cu_2S.

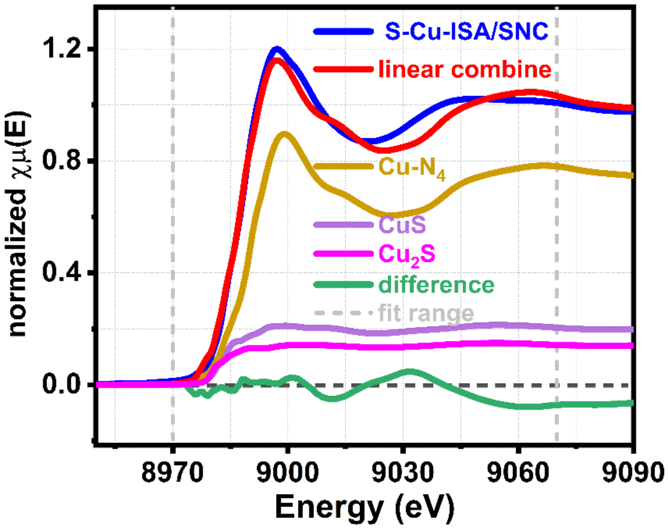

Supplementary Fig. 29. The linear combination fitting of the experimental spectrum of S-Cu-ISA/SNC with the calculated spectrum for CuN_4 and experimental spectra for CuS and Cu_2S.

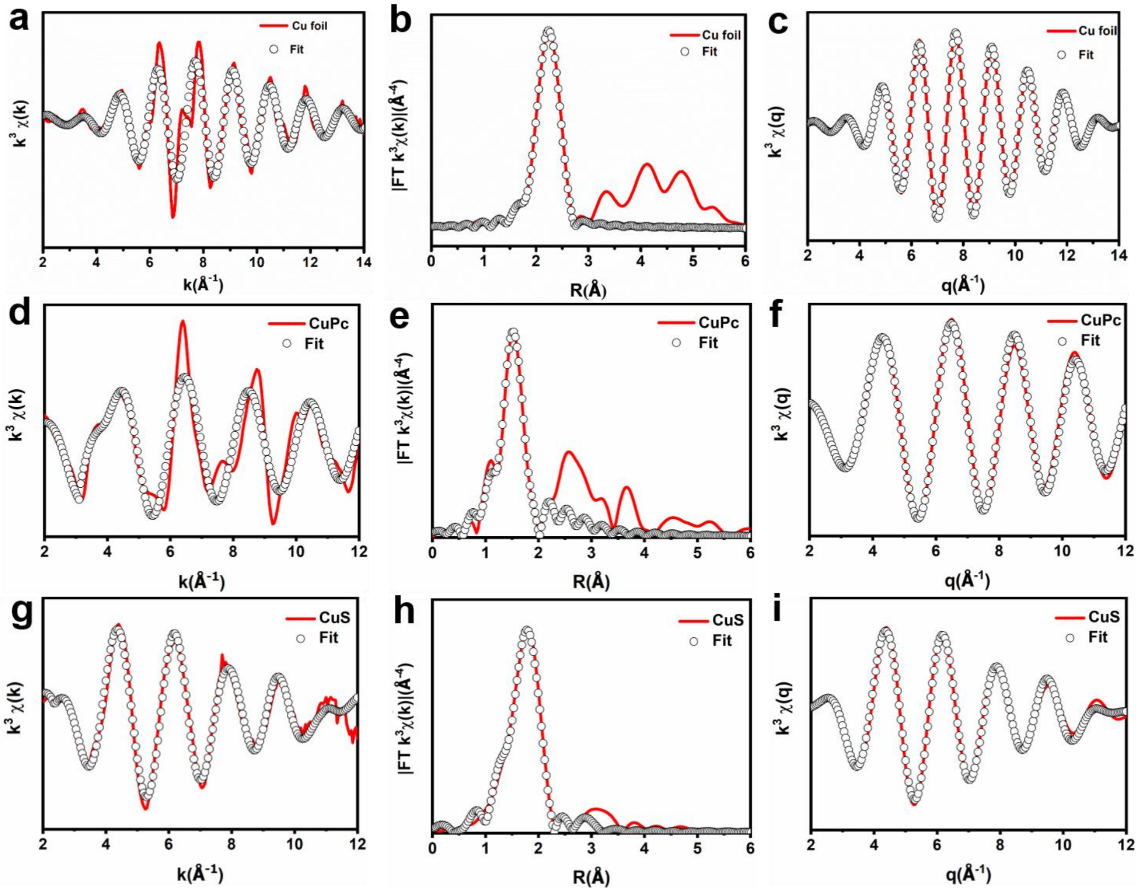

Supplementary Fig. 30. EXAFS fitting curves of Cu foil, CuPc and CuS. (a) the FT-EXAFS fitting curve of Cu foil at Cu K-edge, (b) k space fitting curve and (c) q space fitting curve of Cu foil. (d) the FT-EXAFS fitting curve of CuPc at Cu K-edge, (e) k space fitting curve and (f) q space fitting curve of CuPc. (g) the FT-EXAFS fitting curve of CuS at Cu K-edge, (h) k space fitting curve and (i) q space fitting curve of CuS. The best-fit structural parameters are listed in Supplementary Table 1.

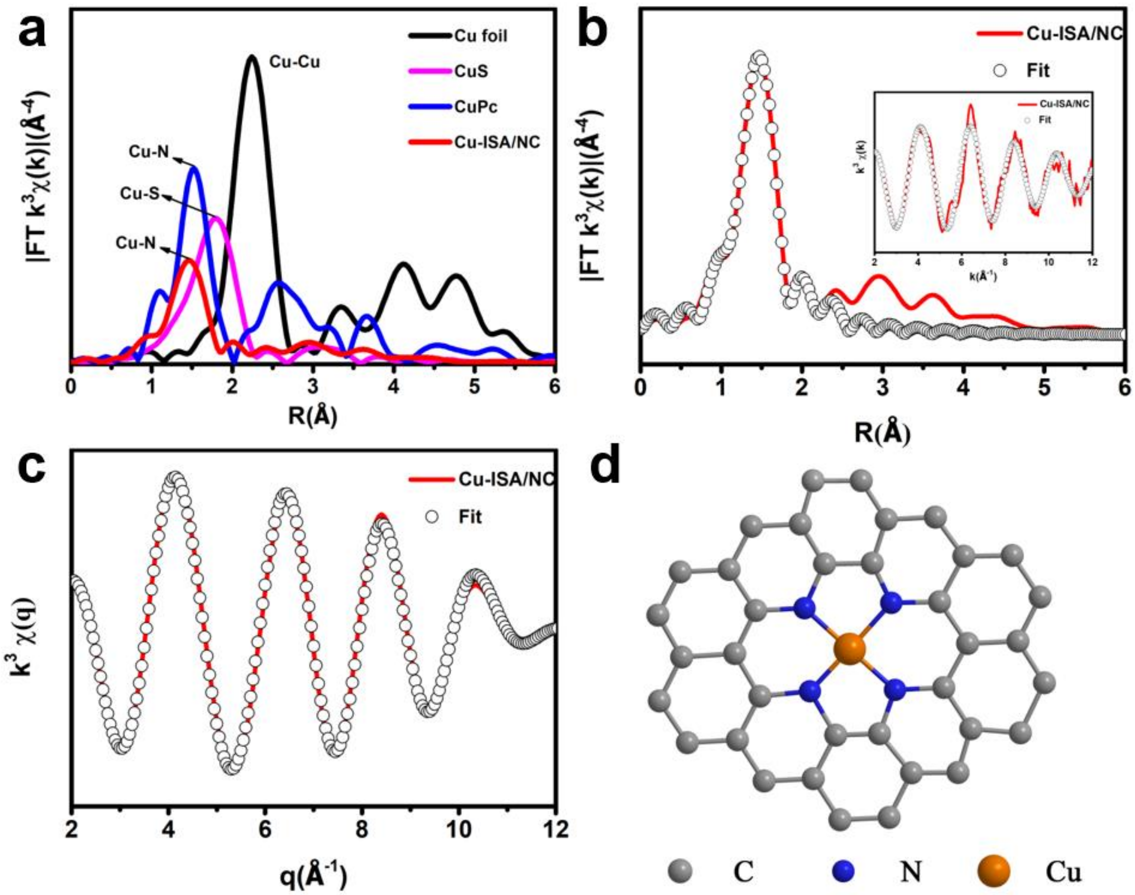

Supplementary Fig. 31. Atomic coordination environment of Cu-ISA/NC. (a) FT k^3-weighted EXAFS spectra of Cu-ISA/NC and the reference samples. (b) FT-EXAFS fitting curves of Cu-ISA/NC and the Corresponding Cu K-edge EXAFS fitting curves. (c) q space fitting curve at Cu K-edge. (d) Atomic interface structure model of Cu-ISA/NC. The best-fit structural parameters are listed in Supplementary Table 1.

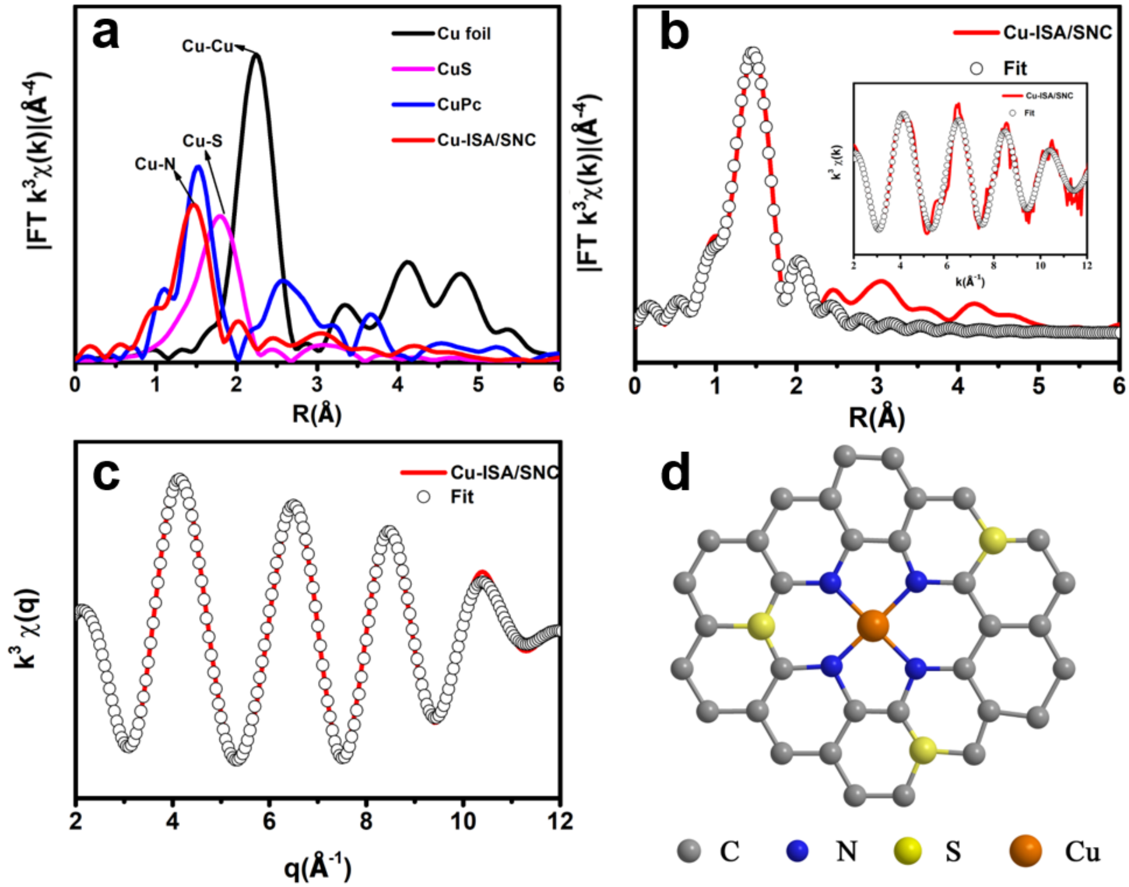

Supplementary Fig. 32. Atomic coordination environment of Cu-ISA/SNC. (a) FT k^3-weighted EXAFS spectra of Cu-ISA/SNC and the reference samples. (b) FT-EXAFS fitting curves of Cu-ISA/SNC and the Corresponding Cu K-edge EXAFS fitting curves. (c) q space fitting curve at Cu K-edge. (d) Atomic interface structure model of Cu-ISA/SNC, in which S atoms are randomly anchored in the carbon matrix. The best-fit structural parameters are listed in Supplementary Table 1.

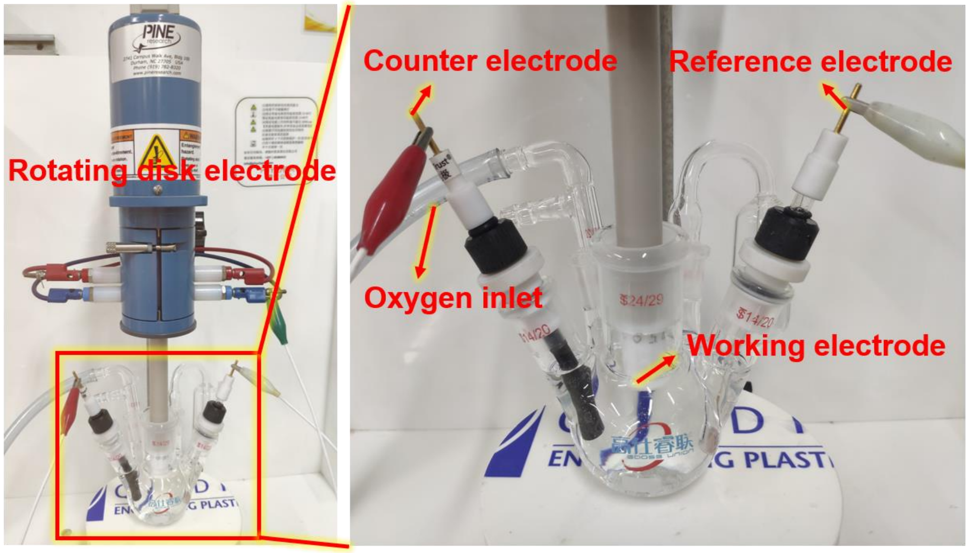

Supplementary Fig. 33. Photograph of the typical three-electrode setup for the electrochemical ORR measurements.

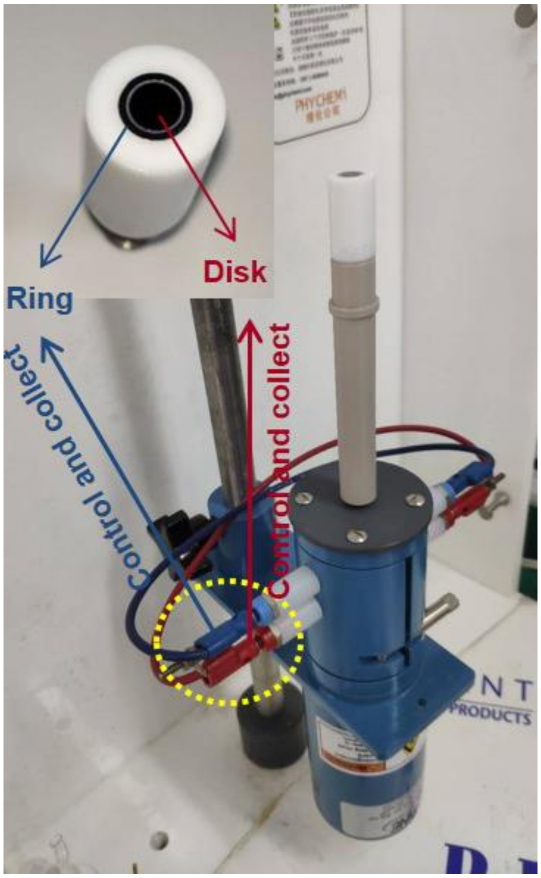

Supplementary Fig. 34. Photograph of rotating ring-disk electrode (PINE). The inset is the enlarged drawings of RRDE. The RRDE consist of Ring and Disk.

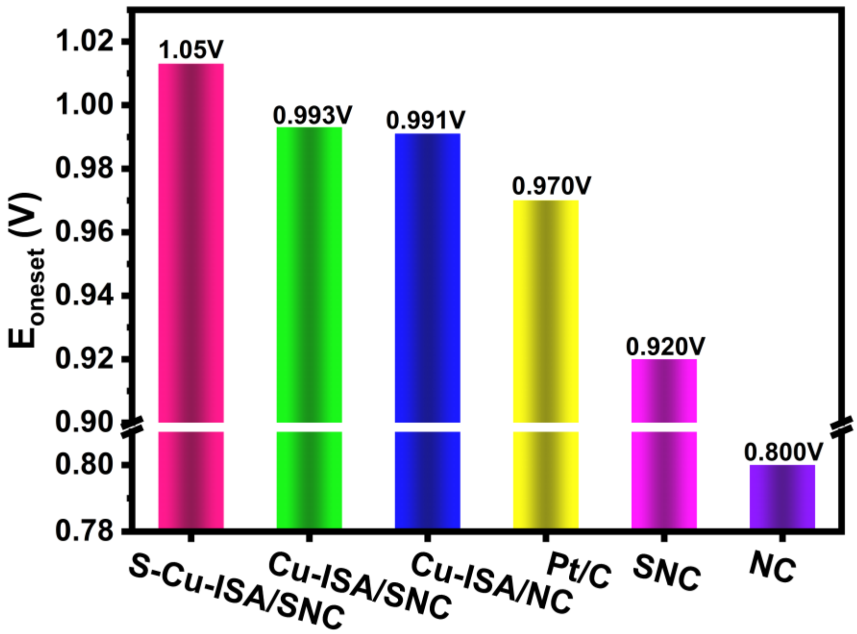

Supplementary Fig. 35. Comparison of E_{oneset} for S-Cu-ISA/SNC with the corresponding reference catalysts.

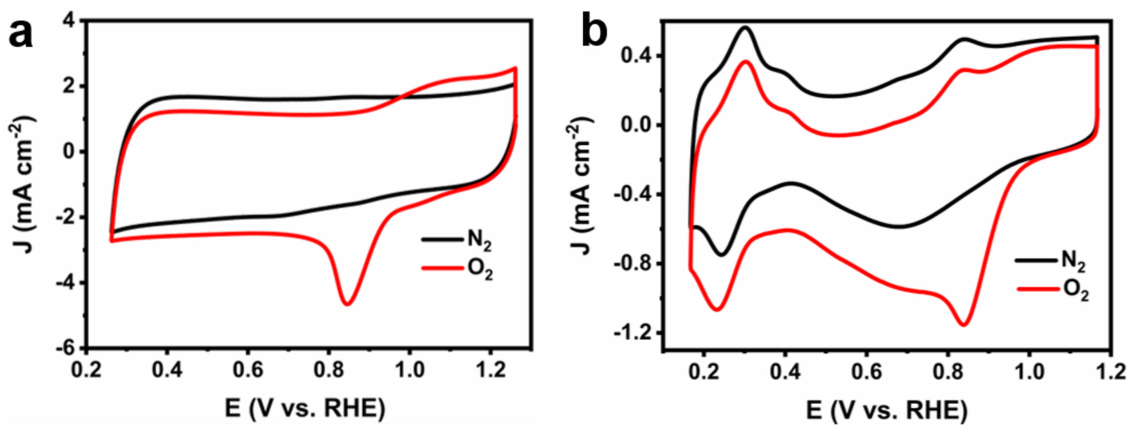

Supplementary Fig. 36. CV test at different atmosphere. (a) CV curves of S-Cu-ISA/SNC in O_2- and N_2-saturated 0.1M KOH electrolyte at a scan rate of 50 mV/s. (b) CV curves of Pt/C in O_2- and N_2-saturated 0.1M KOH electrolyte at a scan rate of 50 mV/s.

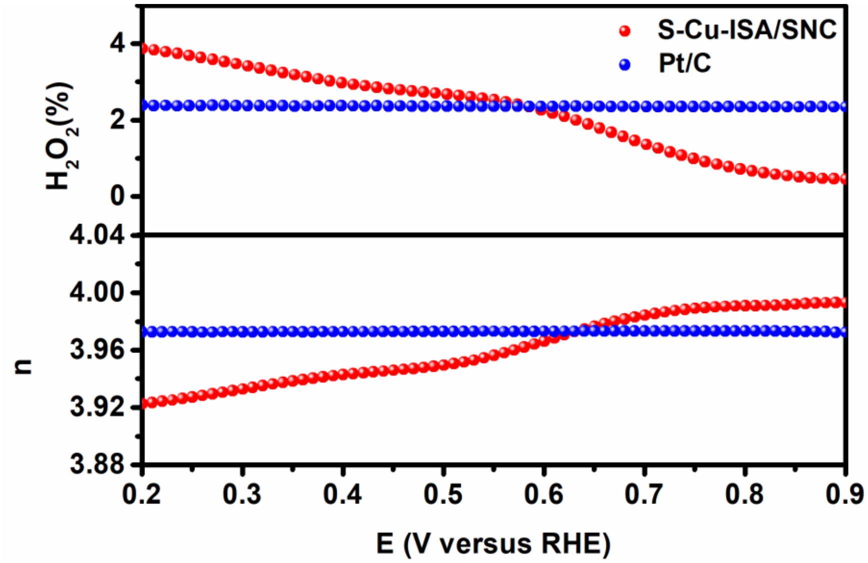

Supplementary Fig. 37. Electron transfer number (*n*, bottom) and H_2O_2 yield (top) versus potential for S-Cu-SAs/SNC and Pt/C.

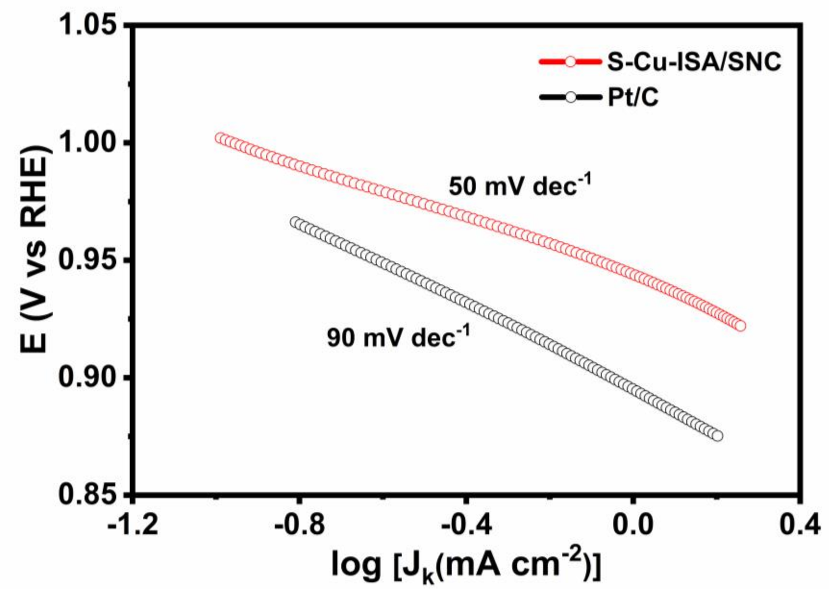

Supplementary Fig. 38. The Tafel plots for S-Cu-ISA/SNC and the Pt/C catalysts.

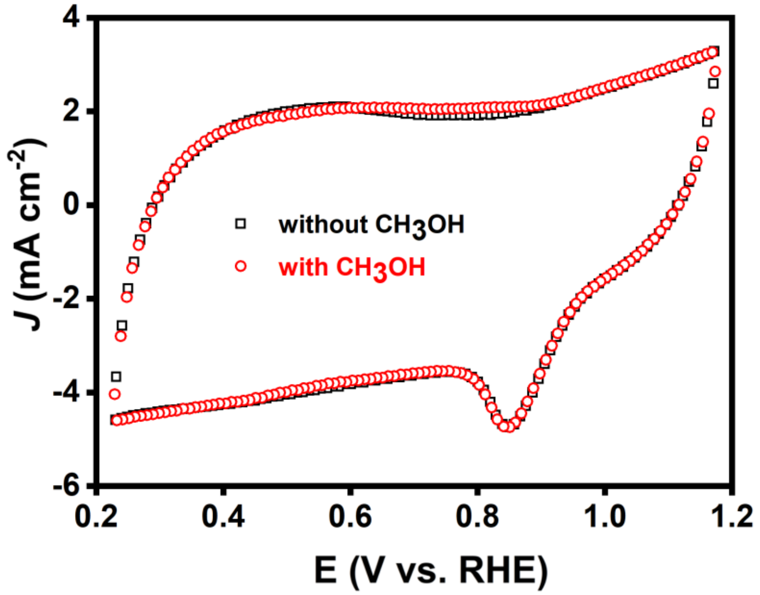

Supplementary Fig. 39. Cyclic voltammetry (CV) data for S-Cu-ISA/SNC in O_2-saturated 0.1 M KOH without and with 0.5 M CH_3OH. There was no obvious change in the current density for the S-Cu-ISA/SNC catalyst after methanol injecting.

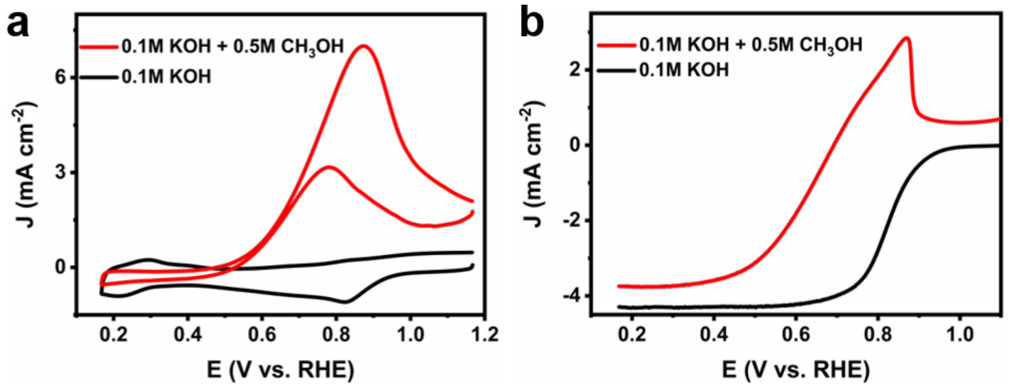

Supplementary Fig. 40. Tolerance test for Pt/C. (a) CV profiles of Pt/C in O_2-saturated 0.1 M KOH solution with (red) or without (black) 0.5 M methanol at a scan rate of 50 mV/s. (b) LSV data of Pt/C in O_2-saturated 0.1 M KOH without and with 0.5 M CH_3OH. The ORR peak disappeared and an obvious peak for the oxidation of methanol was detected in CV for Pt/C.

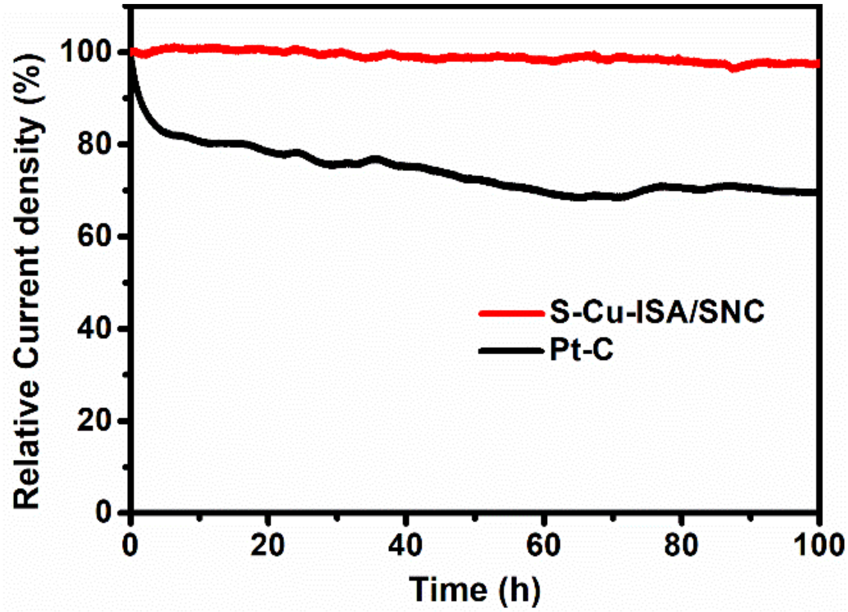

Supplementary Fig. 41. Chronoamperometric response at 0.90 V for S-Cu-ISA/SNC.

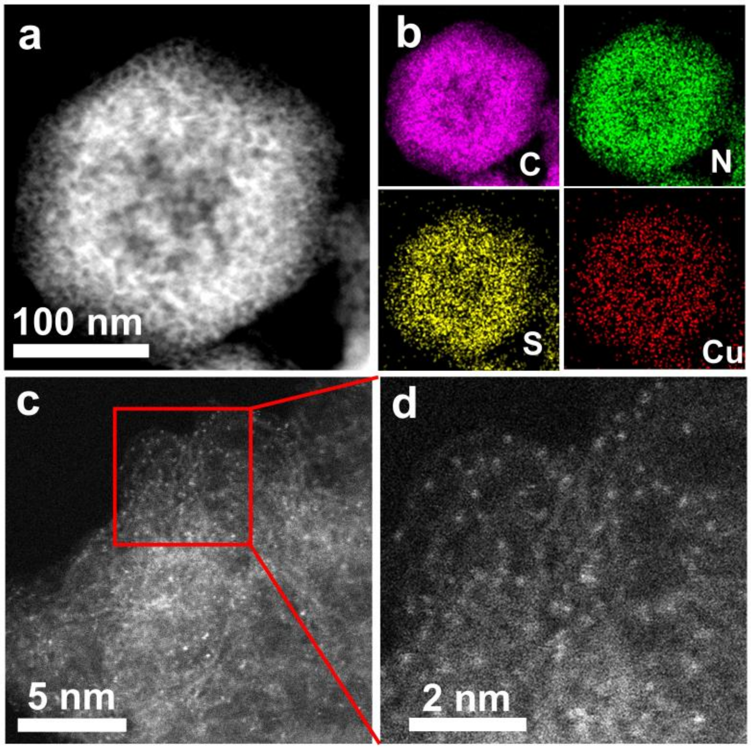

Supplementary Fig. 42. Morphology characterizations of S-Cu-ISA/SNC catalyst after durability test. (a)TEM (b) EDS images, C (pink), N (green), S (yellow) and Cu (red), (c) HAADF-STEM images and (d) magnified images of S-Cu-ISA/SNC catalyst after durability test.

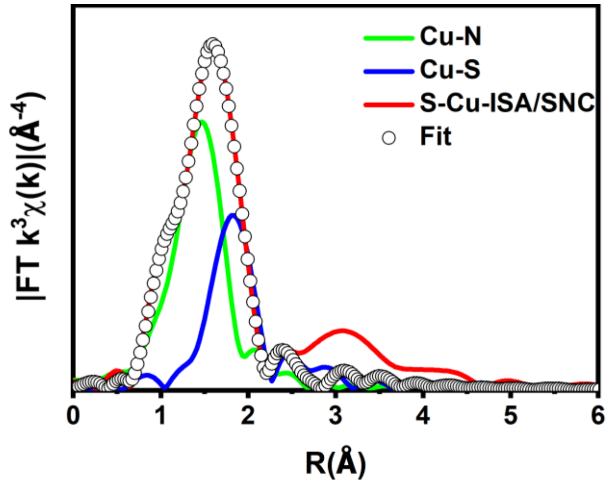

Supplementary Fig. 43. FT-EXAFS fitting curves of S-Cu-ISA/SNC and the Corresponding Cu K-edge EXAFS fitting curves after ORR durability test. The EXAFS fitting revealed that the Cu-SA/SNC were still atomically anchored in the N-doped porous carbon matrix, four-fold coordinated by one S atom and three N atoms.

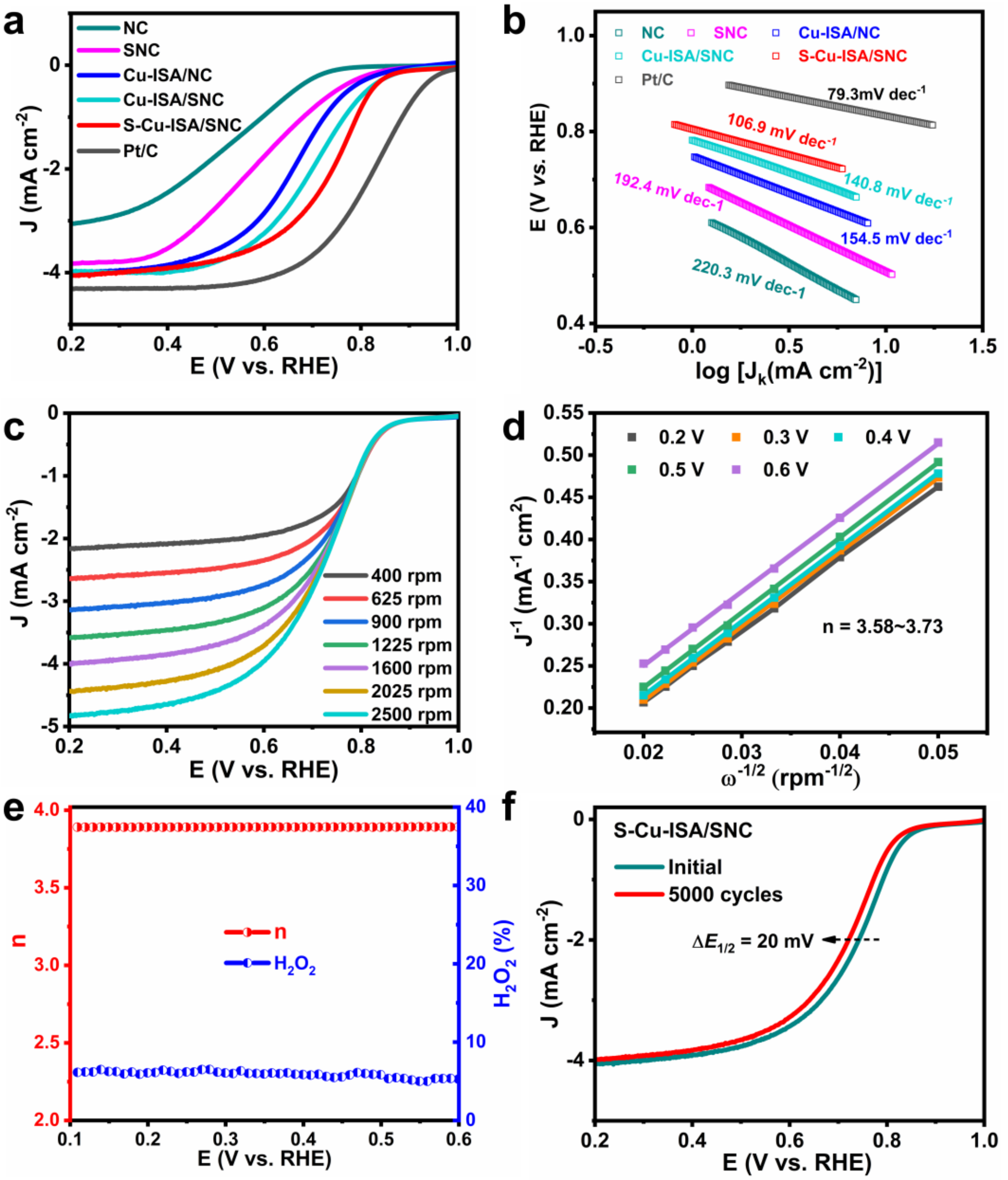

Supplementary Fig. 44. Electrocatalytic ORR performance of S-Cu-ISA/SNC in 0.5M H_2SO_4 solution and 20% Pt/C in 0.1 M $HClO_4$ solution. (**a**) ORR polarization curves for S-Cu-ISA/SNC, Cu-ISA/SNC, Cu-ISA/NC, SNC, NC and 20% Pt/C. (**b**) The Tafel plots for S-Cu-ISA/SNC and the corresponding reference catalysts from the data in panel a. (c) The ORR polarization curves of S-Cu-ISA/SNC at different rotating rates. (d) The K-L plots and electron-transfer numbers for S-Cu-ISA/SNC. (**e**) Electron transfer number (n) (top) and H_2O_2 yield (bottom) *vs.* potential. (f) ORR polarization curves of S-Cu-ISA/SNC before and after 5000 potential cycles. Supplementary Fig. 44a showed the ORR polarization curves of different catalysts. S-Cu-ISA/SNC exhibited the highest

ORR activity among the three Cu-based catalysts (S-Cu-ISA/SNC, Cu-ISA/SNC and Cu-ISA/NC), with onset (E_{onset}) and half-wave ($E_{1/2}$) potentials of 0.86 and 0.74 V, respectively. The $E_{1/2}$ of S-Cu-ISA/SNC was 80 mV lower than that of commercial Pt/C (0.82 V), which was widely used as a benchmark. As we could see, the ORR catalytic activity of S-Cu-ISA/SNC were comparable to SACs of type Fe-N-C reported in the literatures under acid conditions (Supplementary Table 3). The Tafel plots exhibited in Supplementary Fig. 44b confirmed the favorable ORR kinetics of S-Cu-ISA/SNC with low Tafel slope of 106.9 mV dec^{-1}. Koutecky-Levich (K-L) plots of S-Cu-ISA/SNC were derived from linear sweep voltammetry (LSV) curves (Supplementary Fig. 44c). The electron transfer number (n) of S-Cu-ISA/SNC was 3.58-3.73 (Supplementary Fig. 44d), indicating a direct four-electron ORR pathway in acidic conditions. As shown in the Supplementary Fig. 44e, the electron transfer number of S-Cu-ISA/SNC was about 3.90 and the H_2O_2 yield remained below 7%, suggesting its high-efficiency $4e^-$ ORR pathway. Moreover, the accelerated durability of S-Cu-ISA/SNC revealed well stability after 5000 cycles (Supplementary Fig. 44f).

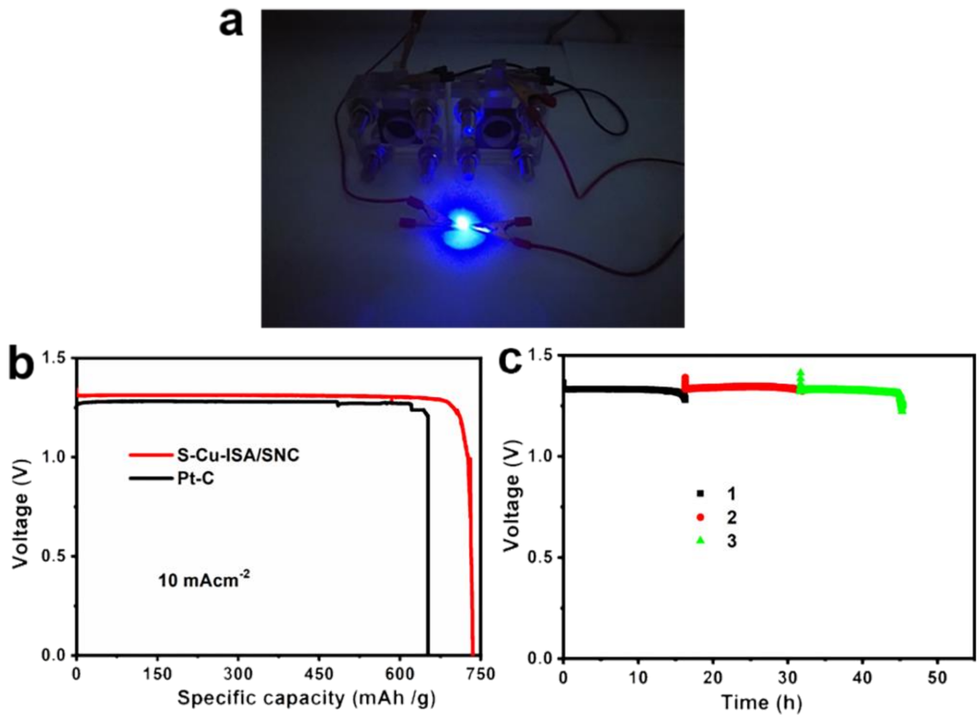

Supplementary Fig. 45. The performance of S-Cu-ISA/SNC-based Zn-air batteries. (a) a photograph showing lamp bulb powered by of S-Cu-ISA/SNC-based Zn-air batteries; (b) The specific capacity of S-Cu-ISA/SNC and Pt/C-based Zn-air batteries at 10 mA cm^{-2} ; (c) Long-term stability of the primary Zn-air battery with S-Cu-ISA/SNC cathode on a current density of 10 mA cm^{-2}. The battery was recharged by re-filling the Zn anode and electrolyte.

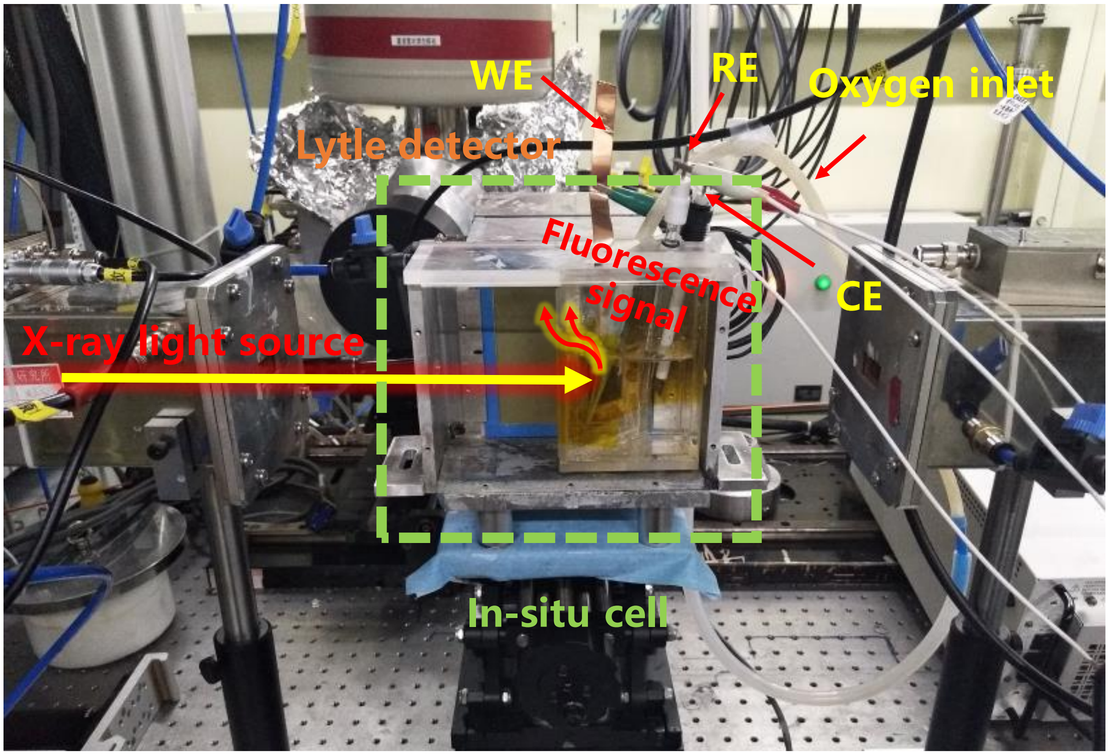

Supplementary Fig. 46. The detail of the *in-situ* X-ray absorption spectroscopy measurement. The device is set up at 14W1 beam line with the support from SSRF, where the X-ray induced fluorescence model is applied. CE, counterelectrode; WE, working electrode; RE, reference electrode.

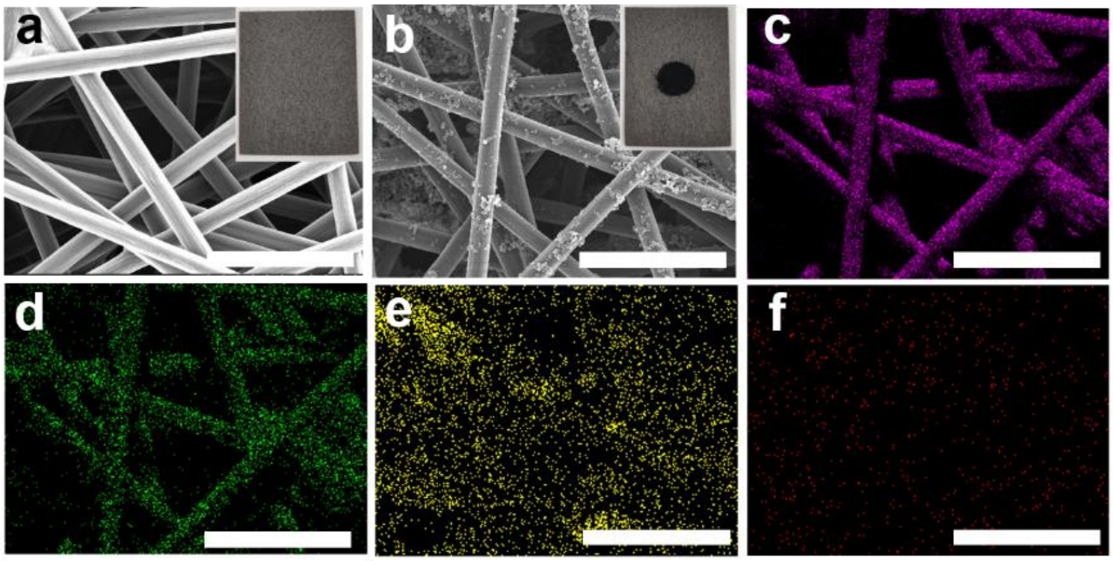

Supplementary Fig. 47. Characterizations of pure carbon paper and working electrode. SEM images for (a) pure carbon paper and (b) working electrode. (c-f) Energy dispersive spectrometry (EDS) mapping of S-Cu-ISA/SNC deposited on carbon cloth, C (pink), N (green), S (yellow) and Cu (red). the scale bar is 50um.

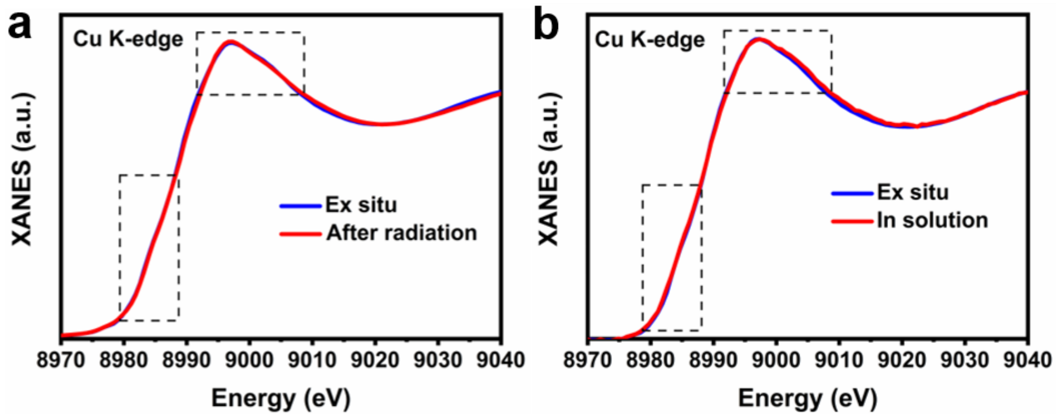

Supplementary Fig. 48. The influence of X-ray radiation and solution. (a) XANES spectra record to monitor X-ray radiation damage on S-Cu-ISA/SNC. It shows that the XANES region with no obvious change. (b) The Cu K-edge XANES spectra for *ex-situ* catalyst and operando catalyst in 0.1 M KOH solution. It can be found that the XANES spectrum for the catalyst in the solution is unchanged in relation to the *ex-situ* XANES data, precluding the adsorption of H_2O molecule.

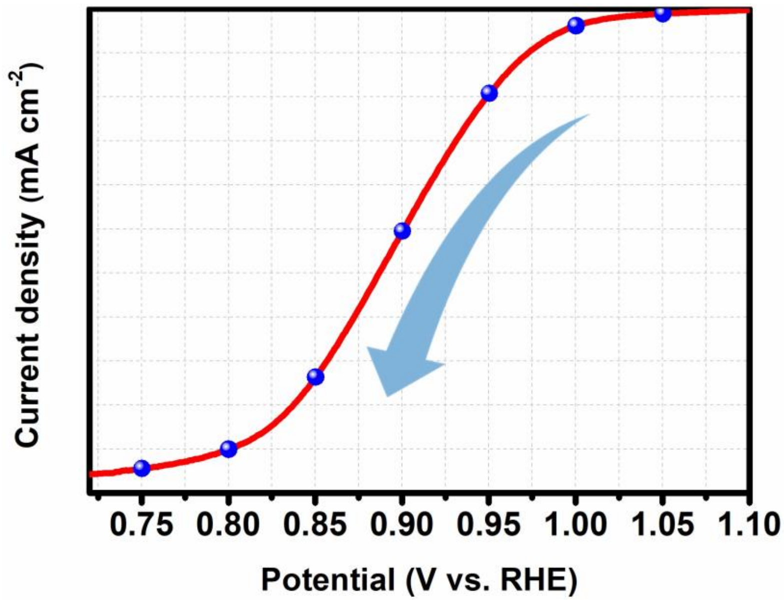

Supplementary Fig. 49. ORR polarization curve for S-Cu-ISA/SNC under *in-situ* XAFS condition (1.05V, 1.00V, 0.95V, 0.90V, 0.85V, 0.80V, 0.75V).

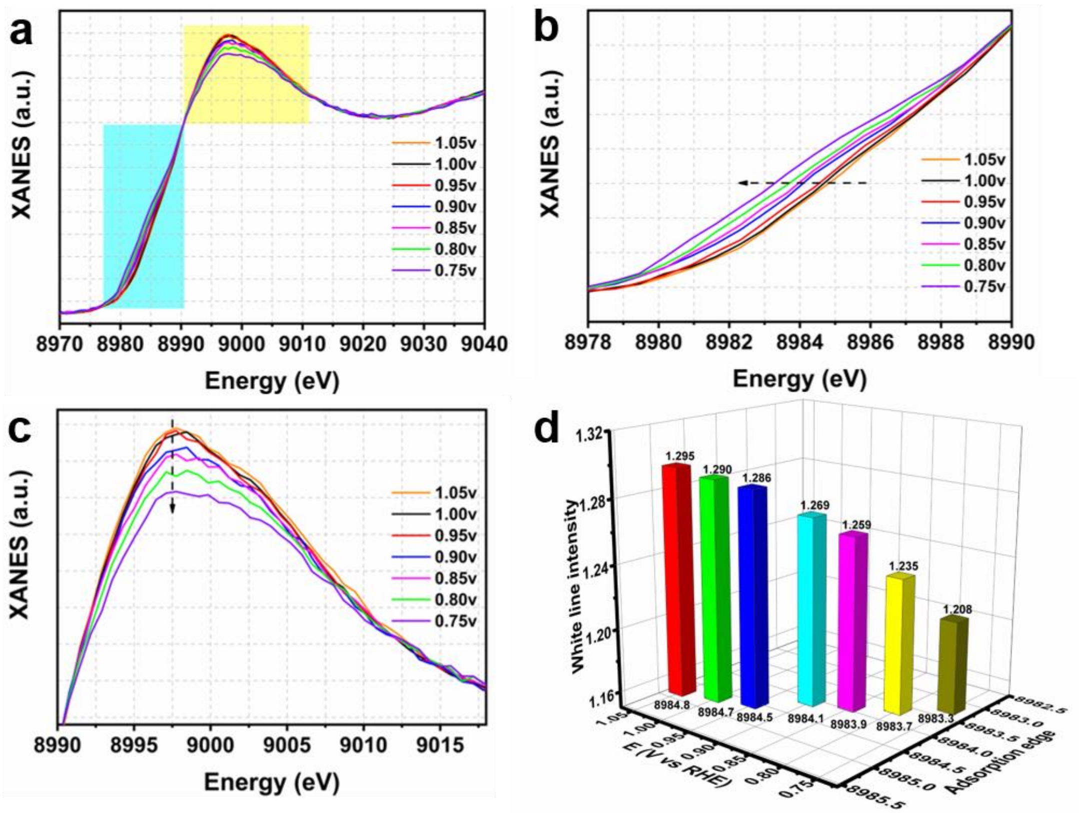

Supplementary Fig. 50. ***In-situ* XAFS characterization of S-Cu-ISA/SNC.** (a) Cu K-edge XANES spectra of S-Cu-ISA/SNC at various potentials during ORR catalysis in O_2-saturated 0.1 M KOH; (b) the absorption edge evolution and (c) the white line peak evolution of S-Cu-ISA/SNC at Cu K-edge under *in-situ* XAFS condition; (d) the histogram which shows the absorption edge position and white line peak intensity evolution of S-Cu-ISA/SNC at Cu K-edge under *in-situ* XAFS condition.

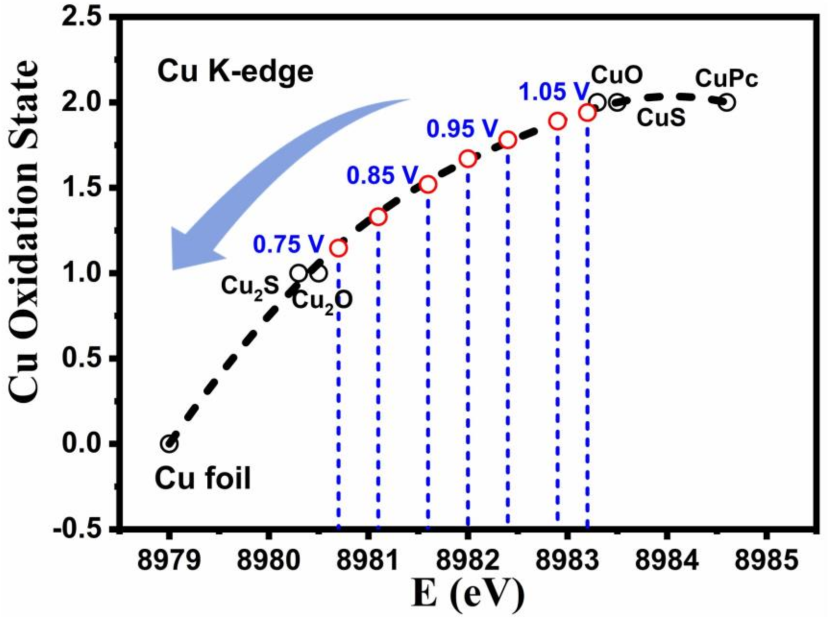

Supplementary Fig. 51. The fitted average oxidation states of Cu from XANES spectra. It was observed that the average valence state of Cu decreased from +1.94 to +1.14 under working conditions (from 1.05 V to 0.75 V *vs.* RHE).

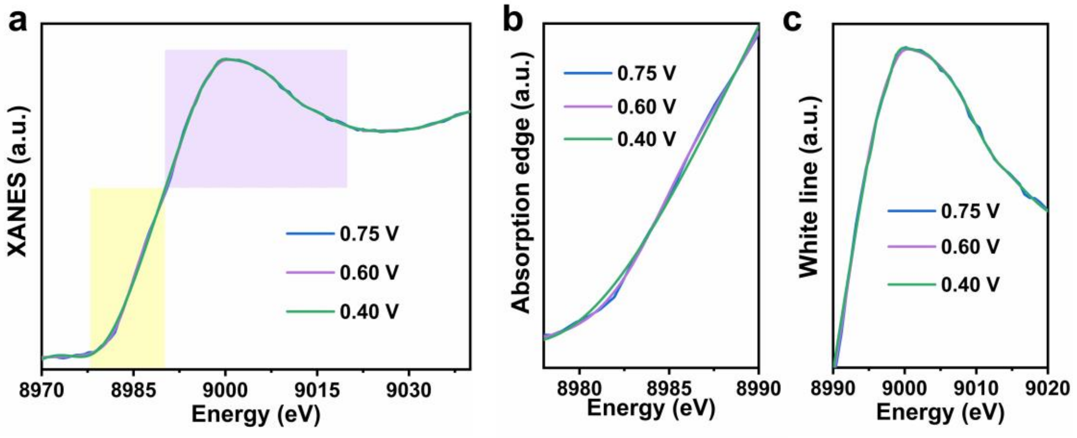

Supplementary Fig. 52. Cu K-edge XANES spectra of S-Cu-ISA/SNC at 0.6 V and 0.4 V compared to that at 0.75 V *vs.* RHE. The XANES spectra at 0.6 V and 0.4 V vs. RHE were recorded (Supplementary Fig. 52a). We could find that the spectra at 0.6 V and 0.4 V vs. RHE showed little change compared to that at 0.75 V (particularly the absorption edge and white line peak as shown in Supplementary Fig. 52b and Supplementary Fig. 52c, respectively), which indicated that the local atomic structure and oxidation state of Cu in S-Cu-ISA/SNC at 0.6 V and 0.4 V during ORR was just the same as that at 0.75 V. This also meant that the average oxidation state of Cu species (Cu-S_1N_3) kept stable (around +1) when the potential was down to 0.75 V, rather than further reduced tends to zero (metallic copper). The extended *in-situ* XAFS measurements gave strong evidences that no Cu particles or clusters form at the applied potentials and also demonstrated that the Cu (+1) sites in S-Cu-ISA/SNC might work as the active centers for ORR.

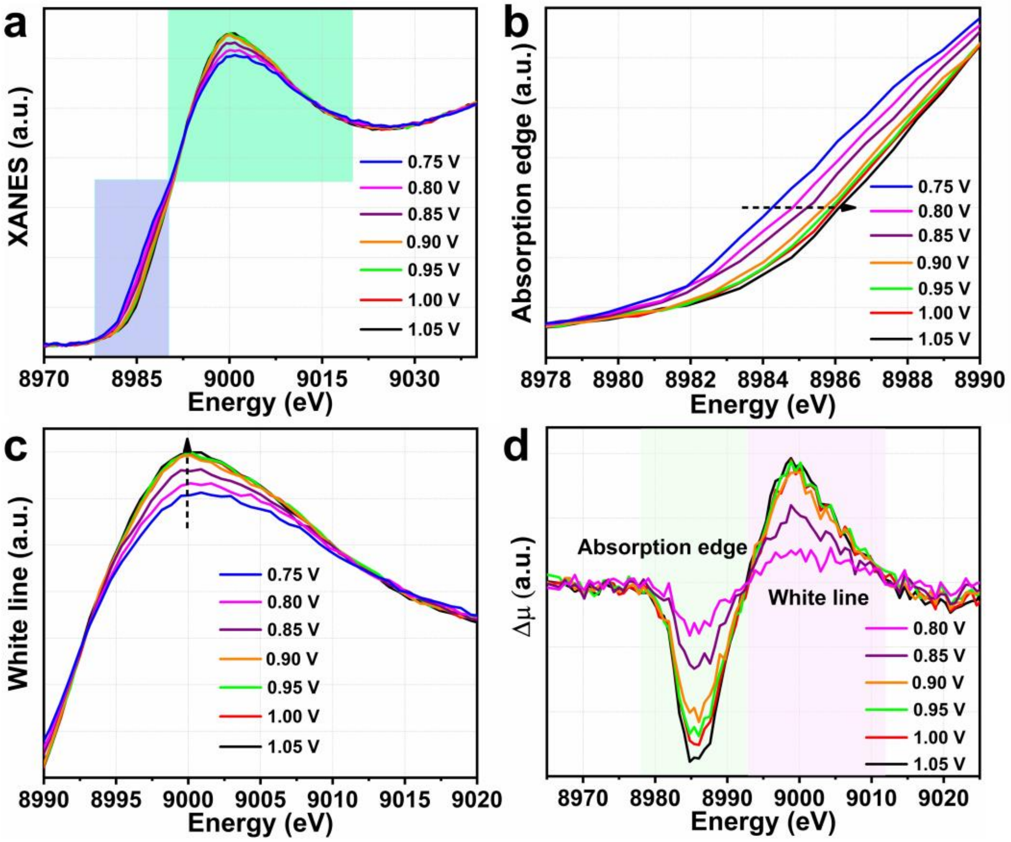

Supplementary Fig. 53. ***In-situ* XAFS characterization of S-Cu-ISA/SNC.** (a) Cu K-edge XANES spectra of S-Cu-ISA/SNC at various potentials during ORR catalysis in O_2-saturated 0.1 M KOH from 0.75 V back to 1.05 V. (b) the absorption edge evolution and (c) the white line peak evolution of S-Cu-ISA/SNC at Cu K-edge under *in-situ* XAFS condition; (d) Differential $\Delta\mu$ XANES spectra obtained by subtracting the normalized spectrum at every potential to the spectrum recorded at 0.75 V *vs.* RHE.

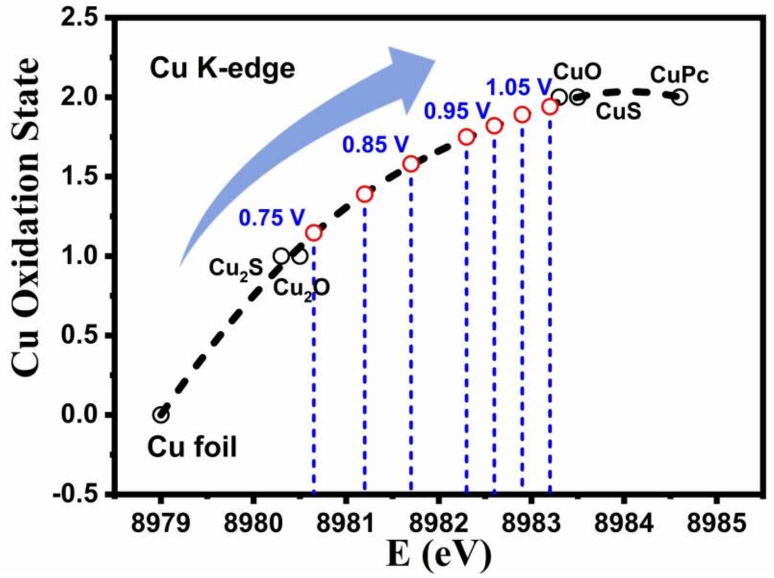

Supplementary Fig. 54. The fitted average oxidation states of Cu from XANES spectra from 0.75 V to 1.05 V *vs.* RHE. It was observed that the average valence state of Cu increase from +1.12 to +1.90 under working conditions.

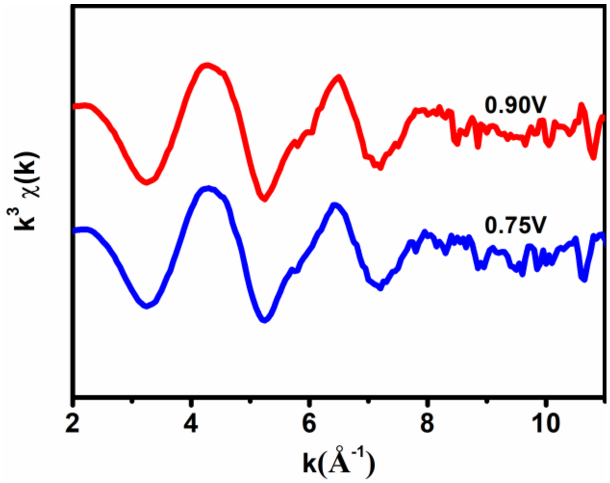

Supplementary Fig. 55. Cu K-edge EXAFS spectra of S-Cu-ISA/SNC at 0.90 V and 0.75 V *vs.* RHE during ORR catalysis in O_2-saturated 0.1 M KOH. Qualitatively, the available information from the EXAFS oscillation is the amplitude, the frequency, and the phase. To a first approximation, amplitude is proportional to coordination number, while frequency is inversely related to bond length. The phase of the EXAFS and the shape of the amplitude envelope provide information about the scatterer.[20, 21]

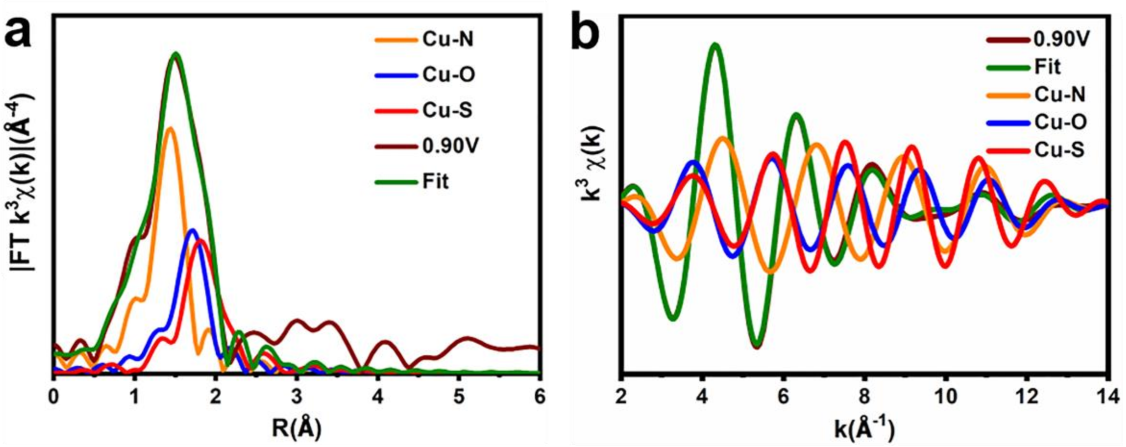

Supplementary Fig. 56. EXAFS fitting at 0.90 V *vs.* RHE. (a) First-shell fitting of EXAFS spectra and (b) corresponding Re($k^3\chi$(k)) oscillations of different fitting paths for the sample at 0.90 V *vs.* RHE. The best-fit structural parameters are listed in Supplementary Table 5.

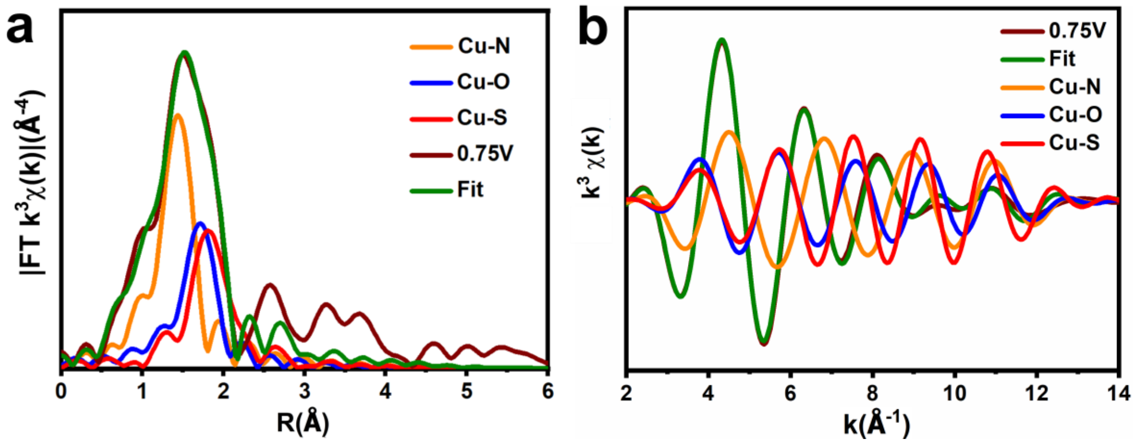

Supplementary Fig. 57. EXAFS fitting at 0.75 V *vs.* RHE. (a) First-shell fitting of EXAFS spectra and (b) corresponding Re($k^3\chi(k)$) oscillations of different fitting paths for the sample at 0.75 V *vs.* RHE. The best-fit structural parameters are listed in Supplementary Table 5.

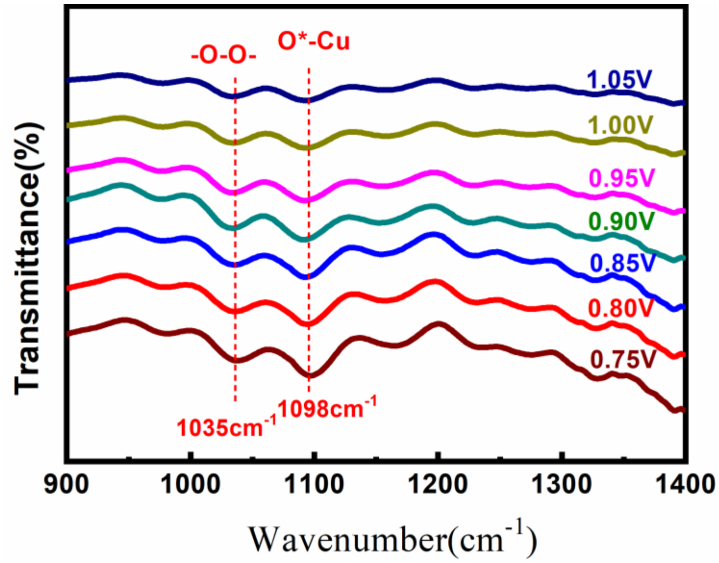

Supplementary Fig. 58. Electrochemical *in-situ* FTIR spectra at different potentials of the S-Cu-ISA/SNC. The superoxide intermediates was detected at 1035 cm^{-1} and the signal of monoxide species was observed at 1098 cm^{-1}.

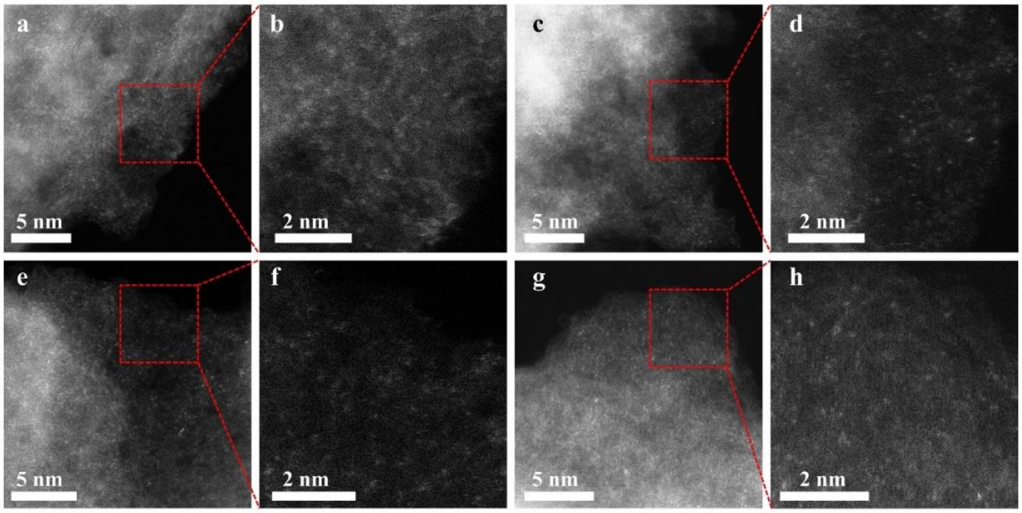

Supplementary Fig. 59. Representative HAADF-STEM images (a, c, e, g) and the enlarged images (b, d, f, h) of the used S-Cu-ISA/SNC catalyst at four different areas after the *in-situ* XAS tests.

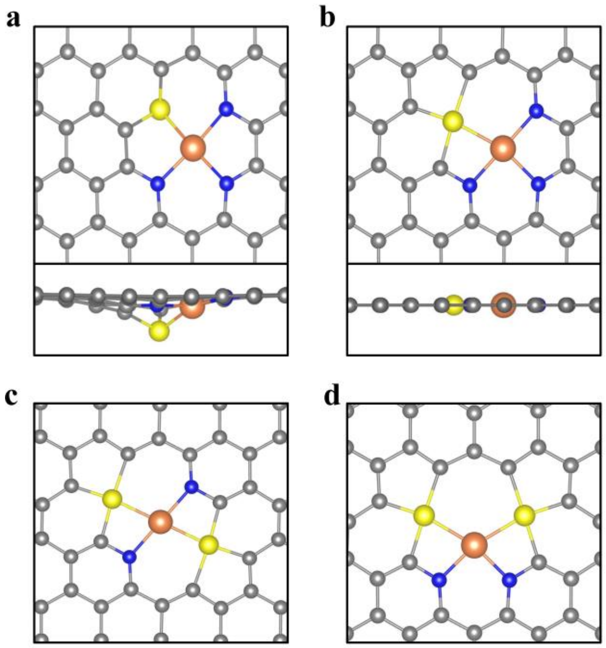

Supplementary Fig. 60. Atomic structures schematics of two kinds of S-doping models and two kinds of Cu-S_2N_2 considered in formation energy calculation. (a) Cu-S_1N_3, in which a doped S atom substitutes only one atom of C or N, and (b) Cu-S_1N_3, in which a doped S atom substitutes two adjacent atoms of C and N. (c) Cu-para-S_2N_2, (d) Cu-ortho-S_2N_2. Gray, blue, orange and yellow balls represent C, N, Cu and S atoms, respectively.

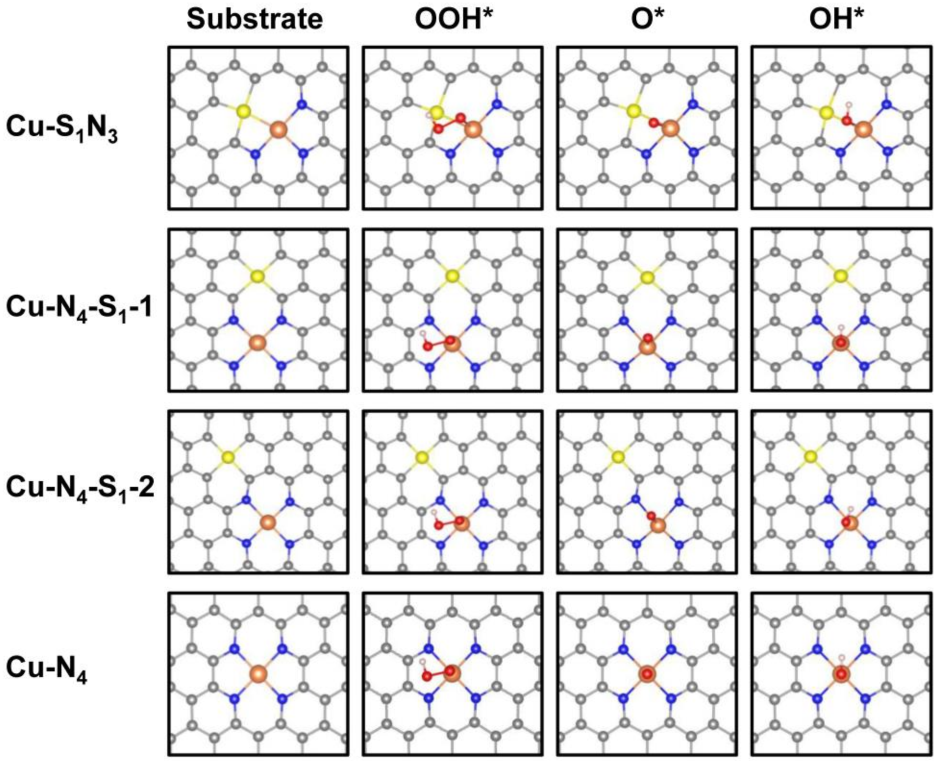

Supplementary Fig. 61. Optimized atomic structures of the ORR intermediates adsorbed on Cu-S_1N_3, Cu-N_4-S_1-1, Cu-N_4-S_1-2 and Cu-N_4 moieties. Gray, blue, orange, yellow, red and white balls represent C, N, Cu, S, O and H atoms, respectively.

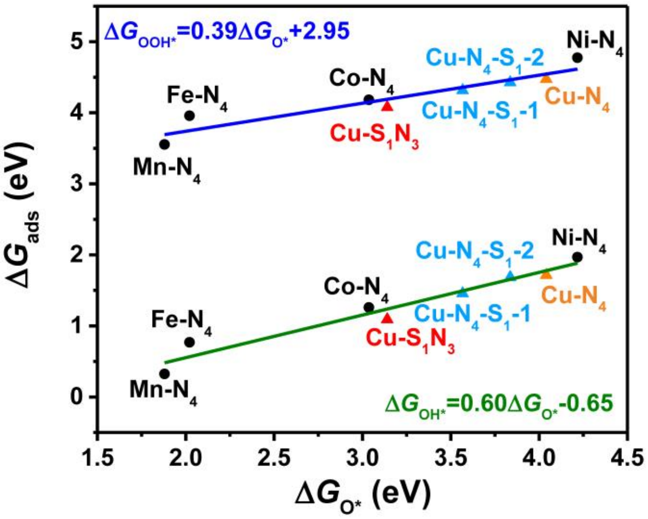

Supplementary Fig. 62. Linear relationship between ΔG_{OH^*}, ΔG_{OOH^*} and ΔG_{O^*} for different Cu-centered moieties embedding in carbon matrix.

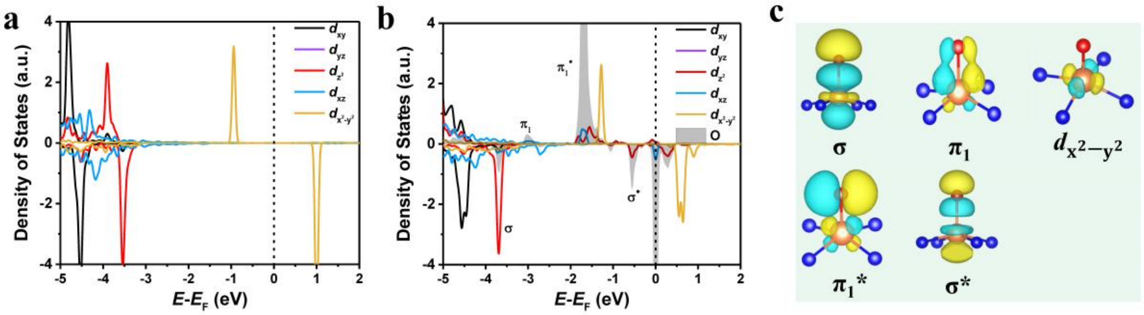

Supplementary Fig. 63. Projected density of states for *d* orbitals of Cu and *p* orbital of O* (a) before and (b) after O* adsorption for $Cu-N_4$. (c) Molecular orbitals of O* adsorbed on $Cu-N_4$. σ and σ* represent the bonding and antibonding between d_{z^2} orbital of Cu and *p* orbital of O, π_1 and π_1* represent the bonding and antibonding between d_{yz}/d_{xz} orbital of Cu and *p* orbital of O.

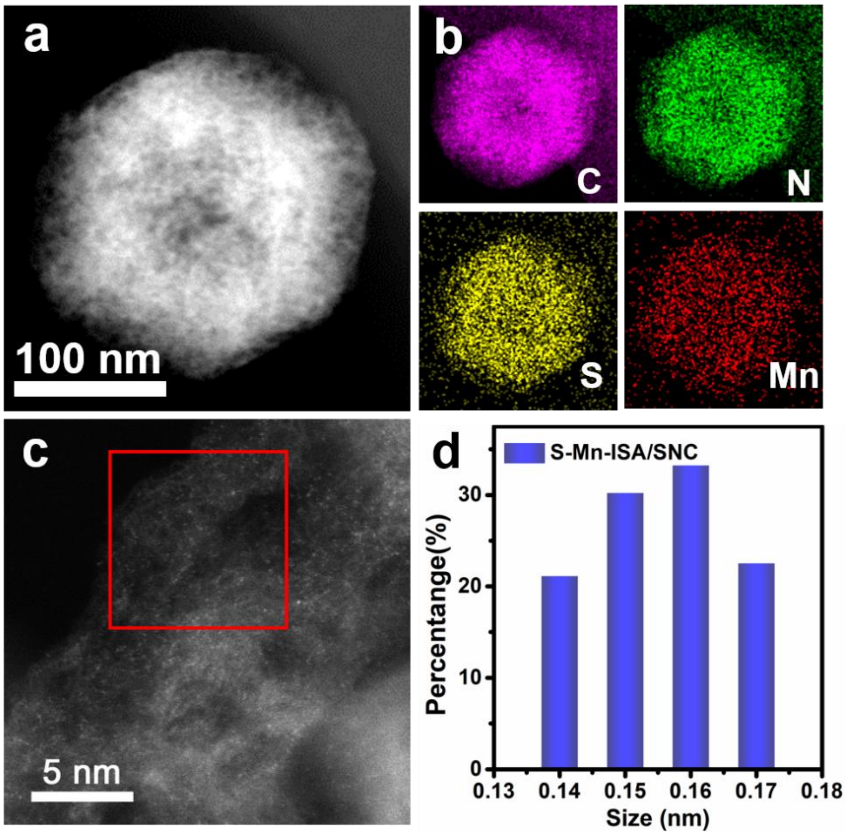

Supplementary Fig. 64. Characterizations of S-Mn-ISA/SNC. (a)TEM (b) EDS images, C (pink), N (green), S (yellow) and Mn (red), (c) HAADF-STEM images and (d) The size distribution of single-atom Mn. All of Mn species in S-Mn-ISA/SNC is less than 0.20 nm, indicating that Mn exists exclusively in atomic dispersion.

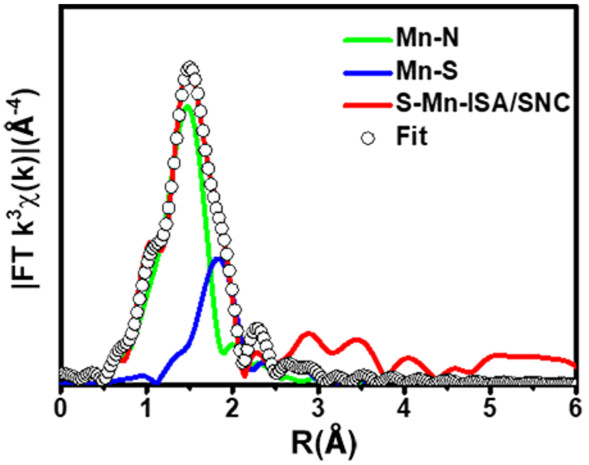

Supplementary Fig. 65. FT-EXAFS fitting curves of S-Mn-ISA/SNC. The best-fit structural parameters are listed in Supplementary Table 10.

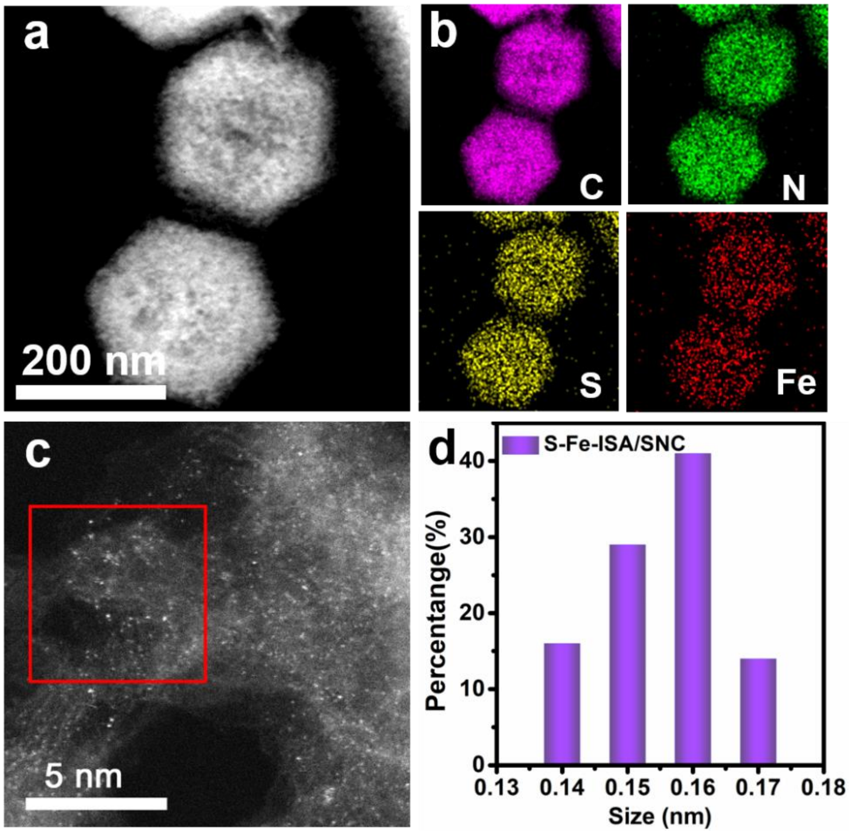

Supplementary Fig. 66. Characterizations of S-Fe-ISA/SNC. (a)TEM (b) EDS images, C (pink), N (green), S (yellow) and Fe (red), (c) HAADF-STEM images and (d) The size distribution of single-atom Fe. All of Fe species in S-Fe-ISA/SNC is less than 0.20 nm, indicating that Fe exists exclusively in atomic dispersion.

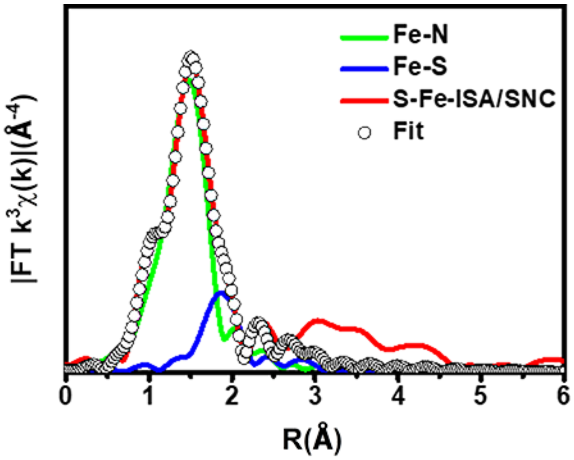

Supplementary Fig. 67. FT-EXAFS fitting curves of S-Fe-ISA/SNC. The best-fit structural parameters are listed in Supplementary Table 10.

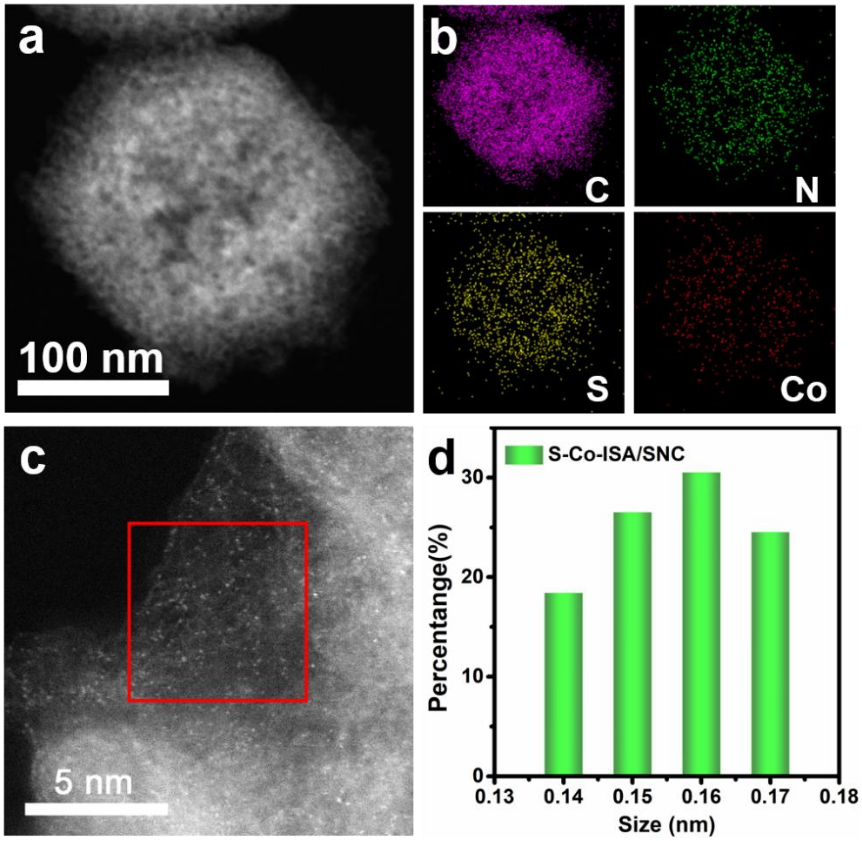

Supplementary Fig. 68. Characterizations of S-Co-ISA/SNC. (a)TEM (b) EDS images, C (pink), N (green), S (yellow) and Co (red), (c) HAADF-STEM images and (d) The size distribution of single-atom Co. All of Co species in S-Co-ISA/SNC is less than 0.20 nm, indicating that Co exists exclusively in atomic dispersion.

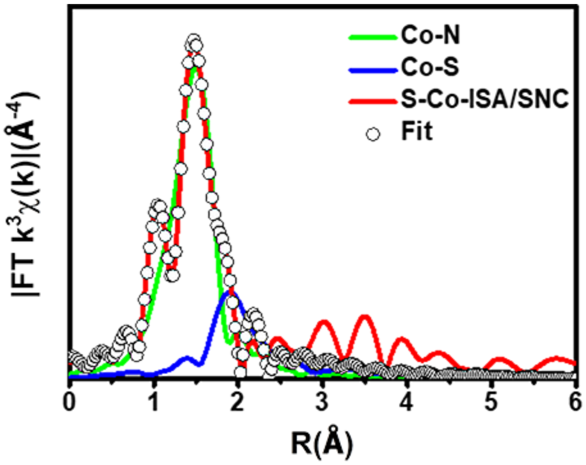

Supplementary Fig. 69. FT-EXAFS fitting curves of S-Co-ISA/SNC. The best-fit structural parameters are listed in Supplementary Table 10.

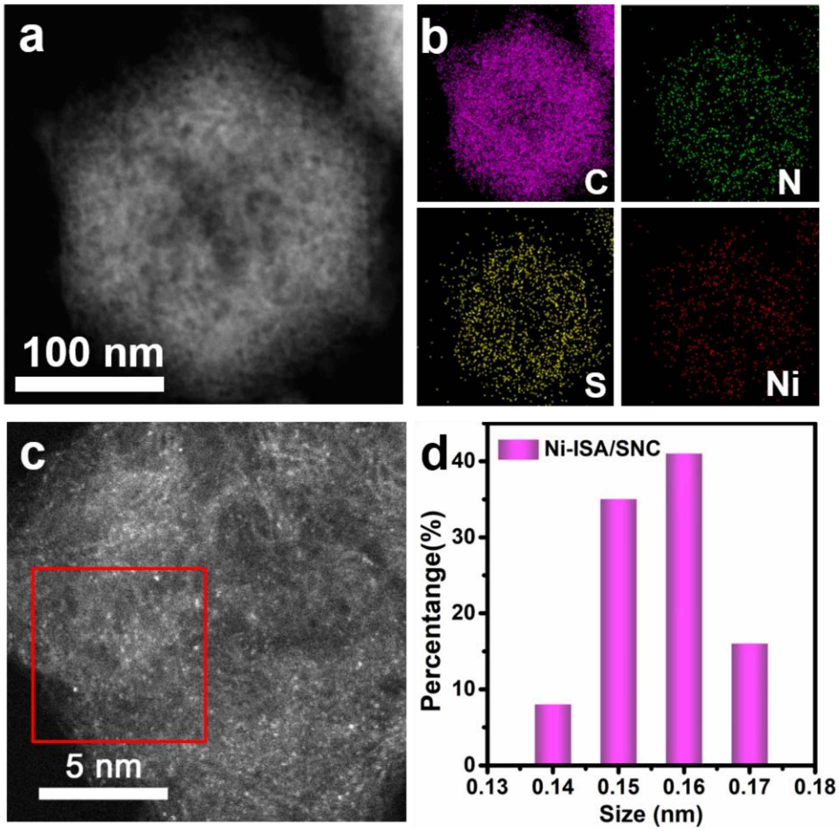

Supplementary Fig. 70. Characterizations of S-Ni-ISA/SNC. (a)TEM (b) EDS images, C (pink), N (green), S (yellow) and Ni (red), (c) HAADF-STEM images and (d) The size distribution of single-atom Ni. All of Ni species in S-Ni-ISA/SNC is less than 0.20 nm, indicating that Ni exists exclusively in atomic dispersion.

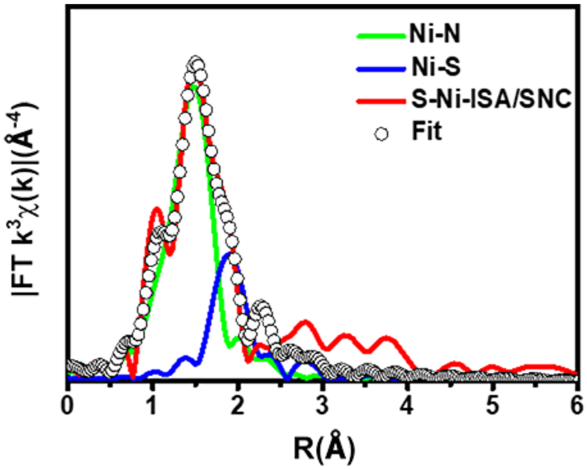

Supplementary Fig. 71. FT-EXAFS fitting curves of S-Ni-ISA/SNC. The best-fit structural parameters are listed in Supplementary Table 10.

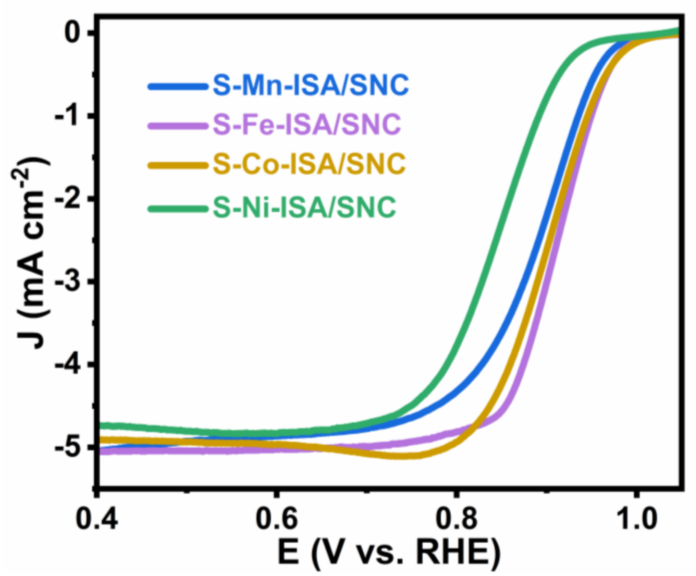

Supplementary Fig. 72. ORR polarization curves for S-Mn-ISA/SNC, S-Fe-ISA/SNC, S-Co-ISA/SNC and S-Ni-ISA/SNC.

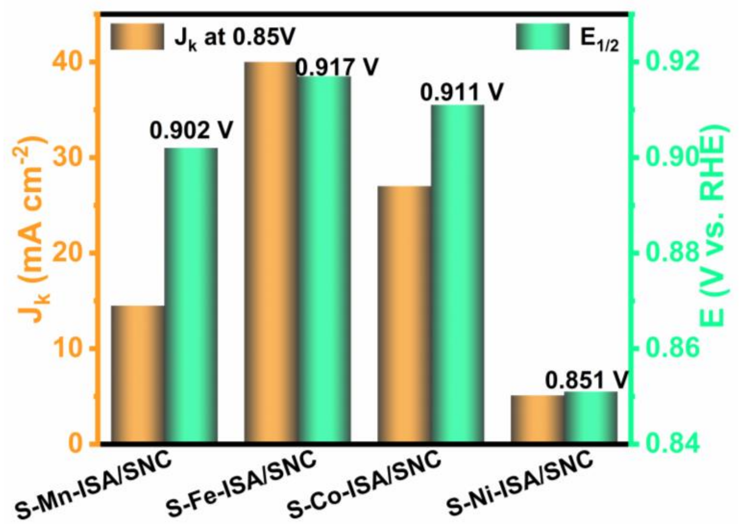

Supplementary Fig. 73. Comparison of J_k at 0.85 V and $E_{1/2}$, for S-Mn-ISA/SNC, S-Fe-ISA/SNC, S-Co-ISA/SNC and S-Ni-ISA/SNC.

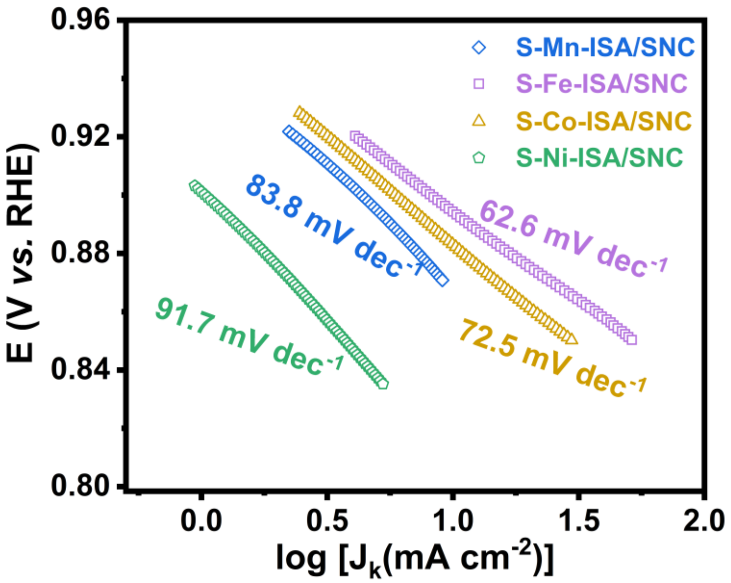

Supplementary Fig. 74. The Tafel plots of S-Mn-ISA/SNC, S-Fe-ISA/SNC, S-Co-ISA/SNC and S-Ni-ISA/SNC. in alkaline media.

Supplementary Tables

Supplementary Table 1. Structural parameters extracted from the Cu K-edge EXAFS fitting. (S_0^2=0.86)

Sample	Scattering pair	CN	R(Å)	$\sigma^2 (10^{-3} Å^2)$	ΔE_0(eV)	R factor
S-Cu-ISA/SNC	Cu-N	2.9	1.98	5.1	1.5	0.005
	Cu-S	1.2	2.32	6.3	2.0	
Cu-ISA/SNC	Cu-N	3.9	1.99	5.9	1.5	0.007
Cu-ISA/NC	Cu-N	4.1	1.99	5.5	1.5	0.006
CuPc	Cu-N	4.0	2.00	4.3	0.5	0.005
CuS	Cu-S	4.2	2.33	5.2	2.0	0.005
Cu foil	Cu-Cu	12*	2.54	8.2	3.5	0.004

S_0^2 is the amplitude reduction factor; CN is the coordination number; R is interatomic distance (the bond length between central atoms and surrounding coordination atoms); σ^2 is Debye-Waller factor (a measure of thermal and static disorder in absorber-scatterer distances); ΔE_0 is edge-energy shift (the difference between the zero kinetic energy value of the sample and that of the theoretical model). R factor is used to value the goodness of the fitting.

* This value was fixed during EXAFS fitting, based on the known structure.

Error bounds that characterize the structural parameters obtained by EXAFS spectroscopy were estimated as N ± 20%; R ± 1%; σ^2 ± 20%; ΔE_0 ± 20%.

Cu-SCN (FT range: 2.0-11.0 $Å^{-1}$; fitting range: 0.8-2.6 Å)

Cu-CNS (FT range: 2.0-11.0 $Å^{-1}$; fitting range: 0.8-2.0 Å)

Cu-CN (FT range: 2.0-11.0 $Å^{-1}$; fitting range: 0.8-2.0 Å)

Cu foil (FT range: 2.0-13.0 $Å^{-1}$; fitting range: 1.2-3.0 Å)

Supplementary Table 2. Comparison of ORR performance between S-CuSAs/SNC and state of the art other non-precious catalysts reported in the literatures under O_2-saturated 0.1 M KOH.

	Electrocatalysts	Onset potential (V *vs.* RHE)	Half-wave potential (V *vs.* RHE)	loading (mg cm-2)	Reference
	S-CuSAs/SNC	1.05	0.918	0.102	This work
1	Co_3O_4/N-rmGO	0.91	0.83	0.17	*Nat. Mater*. **10**, 780 (2011).
2	$Fe_{0.5}$-950	0.97	0.89	0.818	*Nat. Mater*. **14**, 937 (2015)
3	Cu SAs/N-C	~1	0.895	0.09	*Nat. Catal*. **1**, 781–786, (2018).
4	ZnNC	0.92	0.857	0.5	*Nat. Comm.* **10**, 2623 (2019)
5	Fe-SAs/NPS-HC	1.02	0.912	0.510	*Nat. Comm.* **9**, 5422 (2018).
6	Cu-CPG-900	0.978	0.87	0.24	*Nat. Comm.* **5**, 5285 (2014)
7	$ZnMnCoO_4$	1.00	0.88	0.25	*Joule* **2**, 337–348 (2018)
8	Fe SAs-N/C-20	0.97	0.909	0.408	*J. Am. Chem. Soc.***140**, 11594-11598 (2018).
9	Fe@Aza-PON	0.9	0.839	0.159	*J. Am. Chem. Soc.* **140**, 1737−1742 (2018).
10	NCo@CNT-NF700	0.927	0.861	0.2	*J. Am. Chem. Soc.* **140**, 15393−15401, (2108).
11	SA-Fe-HPC	0.96	0.89	-	*Angew. Chem. Int. Ed.* **57**, 9038-9043 (2018).
12	ISA Fe/CN	0.98	0.90	0.408	*Angew. Chem. Int. Ed.* **56**, 6937-6941 (2017).
13	Mn/C-NO	0.94	0.86	0.300	*Adv. Mater*. **30**, 1801732 (2018).

E_{onset}: Onset potential; $E_{1/2}$:Half-wave potential

Supplementary Table 3. Comparison of ORR performance between S-Cu-ISA/SNC and SACs of type Fe-N-C catalysts reported in the literatures under acid electrolyte.

Electrocatalysts	Electrolyte	Onset potential (V *vs.* RHE)	Half-wave potential (V *vs.* RHE)	Limited current (mg cm^{-2})	Reference
S-Cu-ISA/SNC	0.5M H_2SO_4	0.86	0.74	4.06	This work
Fe-SAs/NPS-HC	0.5M H_2SO_4	0.91	0.791	5.01	*Nat. Commun.* **9**, 5422 (2018).
Fe/SNC	0.5M H_2SO_4	0.89	0.77	4.80	*Angew. Chem. Int. Ed.* **56**, 13800-13804 (2017).
SA-Fe/NG	0.5M H_2SO_4	0.90	0.80	5	*PNAS.* **115,** 6626-6631 (2018).
Fe-N-CNF	0.5M H_2SO_4	0.84	0.62	5.2	*Angew. Chem. Int. Ed.* **54**, 8179 (2015).
Fe-ISAs/CN	0.1M $HClO_4$	0.9	0.79	5.8	*Angew. Chem. Int. Ed.* **56**, 6937-6941 (2017).
Fe–N/C-800	0.1M $HClO_4$	0.82	0.6	6.09	*J. Am. Chem. Soc.*, **136**, 11027-11033 (2014).
Fe-CNT/PC	0.1M $HClO_4$	0.95	0.79	5.9	*J. Am. Chem. Soc.* **2016**, *138, 15046-15056.*
CPANI-Fe-NaCl	0.1M $HClO_4$	0.88	0.73	5	*J. Am. Chem. Soc.* **137**, 5414-5420 (2015).

Supplementary Table 4. Comparison of peak power density of different primary Zinc-air batteries reported in literatures.

Catalyst	Catalyst loading ($mgcm^{-2}$)	Peak power density ($mW\ cm^{-2}$)	specific capacity ($mAh\ g^{-1}$)	Reference
S-Cu-ISAs/SNC	1.0	225	735@10 mA cm^{-2}	This work
Zn-N-C-1	0.5	179	683.3@100 mA cm^{-2}	*Angew. Chem.* **27**, 1700802 (2019).
NCNT/CoO-NiO-NiCo	0.53	-	545@10 mA cm^{-2}	*Angew. Chem. Int. Ed.* **54**, 9654-9658 (2015).
NC-Co_3O_4-90	-	82	387.2@20 mA cm^{-2}	*Adv. Mater.* **29**, 1704117 (2017).
Cu-N@C	0.4	210	-	*Energy Environ. Sci.* **9**, 3736 (2016).
$ZnCo_2O_4$/N-CNT	2	82.3	428.47@10 mA cm^{-2}	*Adv. Mater.* **28**, 3777-3784 (2016).
FeNC-S-Fe_xC/Fe	0.2	149.4	663@10 mA cm^{-2}	*Adv. Mater.* **30**, 1804504 (2018).
S,N-Fe/N/CCNT	1.25	102.7	-	*Angew. Chem. Int. Ed.* **56**, 610-614 (2017).
Mn/C-NO	2	120	-	*Adv. Mater.* **30**, 1801732 (2018).
NPMC-1000	0.5	55	735@5 mA cm^{-2}	*Nat. Nanotechnol.* **10**, 444 (2015).
Free-standing Co SA@NCF/CNF	1.2	-	532@6.25 mA cm^{-2}	*Adv. Mater.* 1808267 (2019).
Co-POC	0.1	78	-	*Adv. Mater.* 1900592 (2019).
Mn_3O_4/graphene	2	120	-	*Energy Environ. Sci.* **4**, 4148-4154 (2011).

Supplementary Table 5. Structural parameters extracted from the Cu K-edge EXAFS fitting. (S_0^2=0.86)

Sample	Scattering pair	CN	R(Å)	$\sigma^2 (10^{-3} Å^2)$	ΔE_0(eV)	R factor
S-Cu-ISA/SNC at 0.90 V *vs.* RHE	Cu-N	2.7	1.94	4.9	1.5	0.004
	Cu-O	1.2	1.97	5.3		
	Cu-S	1.1	2.31	6.7	2.0	
S-Cu-ISA/SNC at 0.75 V *vs.* RHE	Cu-N	2.6	1.93	5.6	1.5	0.006
	Cu-O	1.1	1.97	5.3		
	Cu-S	0.9	2.32	6.1	2.0	

S_0^2 is the amplitude reduction factor; CN is the coordination number; R is interatomic distance (the bond length between central atoms and surrounding coordination atoms); σ^2 is Debye-Waller factor (a measure of thermal and static disorder in absorber-scatterer distances); ΔE_0 is edge-energy shift (the difference between the zero kinetic energy value of the sample and that of the theoretical model). R factor is used to value the goodness of the fitting.

Error bounds that characterize the structural parameters obtained by EXAFS spectroscopy were estimated as N ± 20%; R ± 1%; σ^2 ± 20%; ΔE_0 ± 20%.

S-Cu-ISA/SNC at 0.90 V *vs.* RHE (FT range: 2.0-11.0 $Å^{-1}$; fitting range: 0.4-2.6 Å)

S-Cu-ISA/SNC at 0.75 V *vs.* RHE (FT range: 2.0-11.0 $Å^{-1}$; fitting range: 0.4-2.6 Å)

Supplementary Table 6. Atomic radius (r) and electronegativity (χ) of different elements.

Element	r (Å)	χ
C	0.77	2.55
N	0.71	3.04
Cu	1.28	1.91
S	1.04	2.58

Supplementary Table 7. Formation energy ΔE_{form} of different Cu-centered moieties embedding in carbon matrix.

Sample	Structure	ΔE_{form} (eV)
S-Cu-ISA/SNC	Cu-S_1N_3	6.95
	Cu-para-S_2N_2	10.07
	Cu-ortho-S_2N_2	10.58
Cu-ISA/SNC	Cu-N_4-S_1-1	9.03
	Cu-N_4-S_1-2	8.30
Cu-ISA/NC	Cu-N_4	3.33

As for Cu-centered moieties in theoretical structures with one Cu atom, q S atoms, p N atoms and m C atoms (noted as Cu- N_pS_q-C_m), $\Delta E_{form} = E(\text{Cu- }N_pS_q\text{-}C_m) - E(\text{Cu}) - E(N_2)* p/2 - E(\text{S})*q - E(\text{C})*m$, in which E(Cu- N_pS_q-C_m), E(Cu), $E(N_2)$, E(S) and E(C) represent the calculated energy of Cu- N_pS_q-C_m, the Cu atom in bulk Cu, the N_2 molecular, the S atom in S_8 molecular, the C atom in graphene, respectively.

Supplementary Table 8. Reaction free energy (eV *vs.* RHE) of elementary steps for ORR at U_{RHE} = 0V and valence electron number of Cu from Bader charge analysis V_{Cu} for different Cu-centered moieties embedding in carbon matrix.

Sample	structure	ΔG_{OOH*} (eV)	ΔG_{O*} (eV)	ΔG_{OH*} (eV)	V_{Cu}
S-Cu-ISA/SNC	Cu-S_1N_3	4.08	3.14	1.09	10.28
Cu-ISA/SNC	Cu-N_4-S_1-1	4.32	3.57	1.46	10.00
	Cu-N_4-S_1-2	4.43	3.84	1.68	10.03
Cu-ISA/NC	Cu-N_4	4.47	4.04	1.71	9.97

Supplementary Table 9. Metal acetylacetonates feed in synthesis and metal concentration of S-M-ISA/SNC determined by ICP-OES.

Sample	$M(acac)_x$	Metal content/at %	Temperature/℃
S-Mn-ISA/SNC	$Mn(acac)_2$	0.76	950
S-Fe-ISA/SNC	$Fe(acac)_3$	1.11	950
S-Co-ISA/SNC	$Co(acac)_3$	0.87	950
S-Ni-ISA/SNC	$Ni(acac)_2$	0.81	950

The content of Mn, Fe, Co and Ni was measured through ICP-OES.

Supplementary Table 10. Structural parameters extracted from the Mn, Fe, Co and Ni K-edge EXAFS fitting. (S_0^2=0.86)

Sample	Scattering pair	CN	R(Å)	$\sigma^2(10^{-3}Å^2)$	ΔE_0(eV)	R factor
S-Mn-ISA/SNC	Mn-N	2.3	1.91	6.8	1.0	0.008
	Mn-S	1.2	2.30	6.3	2.0	
S-Fe-ISA/SNC	Fe-N	2.9	2.02	5.9	0.5	0.005
	Fe-S	1.3	2.31	7.3	1.5	
S-Co-ISA/SNC	Co-N	3.0	1.93	6.2	1.5	0.008
	Co-S	0.9	2.32	6.5	2.0	
S-Ni-ISA/SNC	Ni-N	3.1	1.95	4.9	0.5	0.007
	Ni-S	1.0	2.33	5.7	2.0	

S_0^2 is the amplitude reduction factor; CN is the coordination number; R is interatomic distance (the bond length between central atoms and surrounding coordination atoms); σ^2 is Debye-Waller factor (a measure of thermal and static disorder in absorber-scatterer distances); ΔE_0 is edge-energy shift (the difference between the zero kinetic energy value of the sample and that of the theoretical model). R factor is used to value the goodness of the fitting.

* This value was fixed during EXAFS fitting, based on the known structure.

Error bounds that characterize the structural parameters obtained by EXAFS spectroscopy were estimated as N ± 20%; R ± 1%; σ^2 ± 20%; ΔE_0 ± 20%.

Mn-SCN (FT range: 2.0-11.0 $Å^{-1}$; fitting range: 0.6-2.4 Å)

Fe-SCN (FT range: 2.0-11.0 $Å^{-1}$; fitting range: 0.6-2.4 Å)

Co-SCN (FT range: 2.0-11.0 $Å^{-1}$; fitting range: 0.6-2.4 Å)

Ni-SCN (FT range: 2.0-11.0 $Å^{-1}$; fitting range: 0.6-2.4 Å)

Supplementary Notes

Supplementary Note. 1 | *In-situ* environmental microscopic study

The *in-situ* environmental microscopic study is performed on a Titan ETEM microscope (FEI) operated at 300 kV equipped with an image Cs-corrector. The sampling nanoparticles are diluted in isopropanol and dispersed with ultrasonic. Then the solution is cast on a MEMS in situ heating chip, blow dried with dry air and mounted on a functional TEM holder (DENSsolutions, wildfire). To avoid cross pollution, both the chip and the sample holder were plasma cleaned by 30 min. After casting, the chip and the holder were again cleaned by UV for 5 min under 35 mbar vacuum to remove the residual organics. The gaseous environment was created inside the ETEM column with pure Ar (99.9995%) and heated by the MEMS heater integrated on the chip.

Supplementary Note. 2 | Soft-XAS and XPS measurements

XPS experiments were performed at the Photoemission Endstation at the BL10B beamline in the National Synchrotron Radiation Laboratory (NSRL). The XANES spectra (C K-edge, N K-edge and S L-edge) were measured at beamline BL12B of National Synchrotron Radiation Laboratory (NSRL). The Cu L-edge XANES spectra of Cu-SA/SNC were collected at the BL11A beamline of National Synchrotron Radiation Research Center (NSRRC). The XANES spectra of S K-edge were recorded at the 4B7A station in Beijing Synchrotron Radiation Facility in TEY mode. The samples were deposited onto double-sided carbon tape for X-ray spectroscopy.

Supplementary Note. 3 | Identification of S-doping way in isolated single Cu-centered moiety embedding in the carbon matrix.

To identify the S-doping way in isolated single Cu-centered moieties embedding in carbon matrix, we tested two doping ways: 1) one S atom substitutes only one atom of C or N; 2) one S atom substitutes two adjacent atoms of C and N (Supplementary Fig. 60). In the first way, the S atom tends to move away from the basal plane since the S atom is much larger than C or N atom (Supplementary Table 6). However, in the second way, the S atom can locate in the basal plane stably when two adjacent atoms of C and N are substituted.[23, 24] So we ues the second model to evaluate the ORR performance of S-doping in the isolated single Cu-centered moiety embedding in the carbon matrix in our calculations.

Supplementary Note. 4 | Preparation of Cu-ZIF-8 and pure ZIF-8

Typically, 1069mg $Zn(NO_3)_2 \cdot 6H_2O$ and 478mg Cupric Acetate Monohydrate ($Cu(acac)_2 \cdot H_2O$) was dissolved in 30 ml of mixture solution (DMF: methanol = 4:1) under sonication for 15 min. After that, 1161mg 2-methylimidazole (2-MeIm) was dispersed into 20 ml of mixture solution (DMF: methanol = 4:1) and was then added into the above solution immediately with vigorous stirring. Subsequently, the mixture solution was sealed for reaction at room temperature for 12 h. The as-prepared product was further collected by centrifugation and washed with methanol and finally dried at 65 °C in a vacuum oven for overnight. Pure ZIF-8 was synthesized by the same method without $Cu(acac)_2$.

Supplementary Note. 5 | Preparation of Cu-ISA/SNC

The obtained SNC powder was firstly dispersed into a mixed solution (DMF: methanol = 4:1) containing $Cu(acac)_2$. After 2 hours ultrasonication along with 10 hours magnetic stirring, the mixture was collected by centrifugation and then dried at 65 °C under vacuum for overnight. Subsequently, the dry powder was ground sufficiently and placed in the quartz tube, followed by heating at 450°C in Ar for 2 hours, and then pyrolyzed in Ar at 950 °C for 4 hours.

Supplementary Note. 6 | Electrochemical data processing

The Tafel slopes were calculated according to the Tafel equation

$$\eta = b \log (j/j_0) \quad (1)$$

based on the LSV curves, where η is the overpotential, b is the Tafel slope, j is the current density, and j_0 is the exchange current density.

The number of electrons transferred (n) and kinetic current density (J_K) during ORR were calculated according to Koutecky-Levich equation:

$$\frac{1}{J} = \frac{1}{J_L} + \frac{1}{J_K} = \frac{1}{B\omega^{0.5}} + \frac{1}{J_K} \quad (2)$$

$$\mathrm{B} = 0.62\mathrm{nF}C_0 D_0^{\frac{2}{3}} V^{-\frac{1}{6}} \quad (3)$$

where J is the measured current density, J_K and J_L are the kinetic and limiting current densities, ω is the angular velocity of the disk, n is the overall number of electrons transferred in oxygen reduction, F is the Faraday constant (96485 C mol^{-1}), C_0 is the bulk concentration of O_2 (1.2×10^{-6} mol cm^{-3}), D_0 is the diffusion coefficient of O_2 in 0.1 M KOH (1.9×10^{-5} cm^2 s^{-1}), and V is the kinematic viscosity of the electrolyte (0.01 cm^2 s^{-1}), and k is the electron transfer rate constant.

The Hydrogen peroxide yield (%H_2O_2) and the electron transfer number (n) were determined by the following equations:

$$\mathrm{H}_2O_2(\%) = \frac{200 \times I_r}{N \times I_d + I_r} \quad (4)$$

$$\mathrm{n} = \frac{4 \times I_d}{I_d + \frac{I_r}{N}} \quad (5)$$

where I_r and I_d are ring and disk currents, and N is collection efficiency (0.37).

Supplementary Note. 7 | XAFS data processing

The acquired EXAFS data were processed according to the standard procedures using the Athena and Artemis implemented in the IFEFFIT software packages. The fitting detail is described below:

The acquired EXAFS data were processed according to the standard procedures using the ATHENA module implemented in the IFEFFIT software packages. The EXAFS spectra were obtained by subtracting the post-edge background from the overall absorption and then normalizing with respect to the edge-jump step. Subsequently, the χ(k) data of were Fourier transformed to real (R) space using a hanning windows (dk=1.0 $Å^{-1}$) to separate the EXAFS contributions from different coordination shells. To obtain the quantitative structural parameters around central atoms, least-squares curve parameter fitting was performed using the ARTEMIS module of IFEFFIT software packages.[22]

The following EXAFS equation was used:

$$\chi(k) = \sum_j \frac{N_j S_o^2 F_j(k)}{k R_j^2} \exp[-2k^2\sigma_j^2] \exp[\frac{-2R_j}{\lambda(k)}] \sin[2kR_j + \phi_j(k)] \qquad (6)$$

S_0^2 is the amplitude reduction factor, $F_j(k)$ is the effective curved-wave backscattering amplitude, N_j is the number of neighbors in the jth atomic shell, R_j is the distance between the X-ray absorbing central atom and the atoms in the jth atomic shell (backscatterer), λ is the mean free path in Å, $\phi_j(k)$ is the phase shift (including the phase shift for each shell and the total central atom phase shift), σ_j is the Debye-Waller parameter of the jth atomic shell (variation of distances around the average R_j). The functions $F_j(k)$, λ and $\phi_j(k)$ were calculated with the ab initio code FEFF8.2. The additional details for EXAFS simulations are given below.

The coordination numbers of model samples (Cu foil) were fixed as the nominal values. The obtained S_0^2 was fixed in the subsequent fitting of Cu single atom samples. While the internal atomic distances R, Debye-Waller factor σ^2, and the edge-energy shift ΔE_0 were allowed to run freely.

Supplementary Note. 8 | The detail of the *in-situ* XAFS measurements

An orgonic glass electrochemical cell was employed for the *in-situ* experiments (Supplementary Fig. 46). The working cell has flat walls with a single circular hole of 1.5 cm in diameter. S-Cu-ISA/SNC coated carbon paper was in contact with a slip of copper with the S-Cu-ISA/SNC layer facing inward. Then KOH solution was poured into the cell. The solutions were not stirred during the experiment. The cell was connected to an electrochemical station by making electrical contact to the copper tape slip that protruded from the side of the working cell. An orgonic glass cap fitted with a reference electrode (Ag/AgCl) was used to cover the cell and to ensure a fixed distance between working and reference electrodes for all experiments. The XAFS spectra were recorded at different positions on the electrode to check the homogeneity of the catalyst. The spectra were recorded on the dry S-Cu-ISA/SNC films at first and then in KOH solution at different potentials. At each potential, 3 scans were recorded at the Cu K-edge. After each potential change, the system was allowed to equilibrate for 20 min before recording a spectrum. A polarization curve of the film under *in-situ* XAFS condition is shown in Supplementary Fig. 49.

Supplementary Note. 9 | The detail of the *in-situ* FTIR measurements

This end station was equipped with a FTIR spectrometer (Bruker 66 v/s) with a KBr beam-splitter and various detectors (here a liquid nitrogen cooled mercury cadmium telluride detector was used) coupled with an infrared microscope (Bruker Hyperion 3000) with a ×16 objective, and can provide infrared spectroscopy measurement with a broad range of 15-4,000 cm^{-1} as well as a high spectral resolution of 0.25 cm^{-1}. The catalyst electrode is tightly pressed against the ZnSe crystal window with a micrometre-scale gap to reduce the loss of infrared light. To ensure the quality of the obtained FTIR spectra, the apparatus was used in a reflection mode with a vertical incidence of infrared light. Each infrared absorption spectrum was acquired by averaging 256 scans at a resolution of 2 cm^{-1}. To guarantee the sustainability of the ORR reactions, O_2 saturation was appropriated via peristaltic pumps with a flow rate of 50 μl h^{-1} during in situ infrared reflection measurements. All infrared spectral acquisitions were carried out after a constant potential was applied to the catalysts electrode for 20 min. The background spectrum of the catalyst electrode was acquired at an open-circuit voltage before each systemic ORR measurement, and the measured potential ranges of the ORR were 1.05-0.75 V *vs.* RHE.

Supplementary References

1. Wang, X. et al. Uncoordinated amine groups of metal–organic frameworks to anchor single Ru sites as chemoselective catalysts toward the hydrogenation of quinoline. *J. Am. Chem. Soc.* **139**, 9419-9422 (2017).
2. Chen, W. et al. Single tungsten atoms supported on MOF-derived N-doped carbon for robust electrochemical hydrogen evolution. *Adv. Mater.* **30**, e1800396 (2018).
3. Jiang, R. et al. Edge-site engineering of atomically dispersed Fe–N_4 by selective C–N bond cleavage for enhanced oxygen reduction reaction activities. *J. Am. Chem. Soc.* **140**, 11594-11598 (2018).
4. Cheng, C. et al. Atomic Fe-N_x coupled open-mesoporous carbon nanofibers for efficient and bioadaptable oxygen electrode in Mg-air batteries. *Adv. Mater.* **30**, 1802669 (2018).
5. Li, S. et al. Active salt/silica-templated 2D mesoporous FeCo-N_x-carbon as bifunctional oxygen electrodes for Zinc-air batteries. *Angew. Chem. Int. Ed.* **57**, 1856-1862 (2018).
6. Qiao, Y. et al. Sulfuration of an Fe-N-C catalyst containing Fe_xC/Fe species to enhance the catalysis of oxygen reduction in acidic media and for use in flexible Zn-air batteries, *Adv. Mater.* **30**, 1804504 (2018).
7. Yang, H. B. et al. Identification of catalytic sites for oxygen reduction and oxygen evolution in N-doped graphene materials: development of highly efficient metal-free bifunctional electrocatalyst. *Sci. Adv.* **2**, e1501122 (2016).
8. Chen, Y. et al. Enhanced oxygen reduction with single-atomic-site iron catalysts for a zinc-air battery and hydrogen-air fuel cell. *Nat. Commun.* **9**, 5422 (2018).
9. Wei, S. et al. Direct observation of noble metal nanoparticles transforming to thermally stable single atoms. *Nat. Nanotechnol.* **13**, 856-861 (2018).
10. Qu, Y., et al. Direct transformation of bulk copper into copper single sites via emitting and trapping of atoms. *Nat. Catal.* **1**, 781-786 (2018).

11. Wu, H. et al. Highly doped and exposed Cu (I)-N active sites within graphene towards efficient oxygen reduction for zinc–air batteries. *Energy Environ. Sci.*, **9**, 3736-3745 (2016).

12. Peng, P., et al. A pyrolysis-free path toward superiorly catalytic nitrogen-coordinated single atom. *Sci. Adv.*, **5**, eaaw2322 (2019).

13. Liu, W., et al. Discriminating catalytically active FeN_x species of atomically dispersed Fe-N-C catalyst for selective oxidation of the C-H bond. *J. Am. Chem. Soc*, **139**, 10790-10798 (2017).

14. Zhang, T., et al. Single atomic Cu-N_2 catalytic sites for highly active and selective hydroxylation of benzene to phenol. *iScience*, **22**, 97-108 (2019).

15. Yang, H. B., et al. Atomically dispersed Ni (I) as the active site for electrochemical CO_2 reduction. *Nat. Energy*, **3**, 140-147 (2018).

16. Shao, X. et al. Wavelet: a new trend in chemistry, *Acc. Chem. Res.* **36**, 276 (2003).

17. Funke, H., Scheinost, A. C. & Chukalina, M. Wavelet analysis of extended x-ray absorption fine structure data. *Phys. Rev. B* **71**, 094110 (2005).

18. Joly, Y. X-ray absorption near-edge structure calculations beyond the muffin-tin approximation. *Phys. Rev. B* **63**, 125120 (2001).

19. Bunău, O. & Joly, Y. Self-consistent aspects of X-ray absorption calculations. *J. Phys. Condens. Matter.* **21**, 345501 (2009).

20. Rehr, J. J. & Albers, R. C. Theoretical approaches to X-ray absorption fine structure. *Rev. Mod. Phys.* **72**, 621-654 (2000).

21. Penner-Hahn, J. E. X-ray absorption spectroscopy in coordination chemistry. *Coord. Chem. Rev.* **190-192**, 1101-1123 (1999).

22. Ravel, B. et al. ATHENA and ARTEMIS: interactive graphical data analysis using IFEFFIT. *Phys. Scr.* 2005, 1007 (2005).

23. Xia, L. *et al.* Sulfur-doped graphene for efficient electrocatalytic N_2-to-NH_3 fixation. *Chem. Commun.* **55**, 3371-3374 (2019).

24. Li, Q. *et al.* Fe Isolated Single Atoms on S, N Codoped carbon by copolymer pyrolysis strategy for highly efficient oxygen reduction reaction. *Adv. Mater.* **30**, 1800588 (2018)
